# Intrinsically-generated fluctuating activity in excitatory-inhibitory networks

**DOI:** 10.1371/journal.pcbi.1005498

**Published:** 2017-04-24

**Authors:** Francesca Mastrogiuseppe, Srdjan Ostojic

**Affiliations:** 1 Laboratoire de Neurosciences Cognitives, INSERM U960, École Normale Supérieure - PSL Research University, Paris, France; 2 Laboratoire de Physique Statistique, CNRS UMR 8550, École Normale Supérieure - PSL Research University, Paris, France; UCL, UNITED KINGDOM

## Abstract

Recurrent networks of non-linear units display a variety of dynamical regimes depending on the structure of their synaptic connectivity. A particularly remarkable phenomenon is the appearance of strongly fluctuating, chaotic activity in networks of deterministic, but randomly connected rate units. How this type of intrinsically generated fluctuations appears in more realistic networks of spiking neurons has been a long standing question. To ease the comparison between rate and spiking networks, recent works investigated the dynamical regimes of randomly-connected rate networks with segregated excitatory and inhibitory populations, and firing rates constrained to be positive. These works derived general dynamical mean field (DMF) equations describing the fluctuating dynamics, but solved these equations only in the case of purely inhibitory networks. Using a simplified excitatory-inhibitory architecture in which DMF equations are more easily tractable, here we show that the presence of excitation qualitatively modifies the fluctuating activity compared to purely inhibitory networks. In presence of excitation, intrinsically generated fluctuations induce a strong increase in mean firing rates, a phenomenon that is much weaker in purely inhibitory networks. Excitation moreover induces two different fluctuating regimes: for moderate overall coupling, recurrent inhibition is sufficient to stabilize fluctuations; for strong coupling, firing rates are stabilized solely by the upper bound imposed on activity, even if inhibition is stronger than excitation. These results extend to more general network architectures, and to rate networks receiving noisy inputs mimicking spiking activity. Finally, we show that signatures of the second dynamical regime appear in networks of integrate-and-fire neurons.

## Author summary

Electrophysiological recordings from cortical circuits reveal strongly irregular and highly complex temporal patterns of in-vivo neural activity. In the last decades, a large number of theoretical studies have speculated on the possible sources of fluctuations in neural assemblies, pointing out the possibility of self-sustained irregularity, intrinsically generated by network mechanisms. In particular, a seminal study showed that purely deterministic, but randomly connected rate networks intrinsically develop chaotic fluctuations due to the recurrent feedback. In the simple and highly symmetric class of models considered in classical works, the transition from stationary activity to chaos is characterized by the behavior of the auto-correlation function and the critical slowing down of fluctuations. Following up on recent works, here we combine analytical and numerical tools to investigate the macroscopic dynamics generated by more realistic models of excitatory and inhibitory rate units. We show that the presence of excitation leads to a strong signature of the onset of chaos in the first-order statistics of the network activity, and that this effect is highly robust with respect to spiking noise. We moreover find that excitation leads to two different types of fluctuating activity at moderate and strong synaptic coupling, even when inhibition dominates. Finally, we test the appearance of analogous dynamical regimes in networks of integrate-and-fire neurons.

## Introduction

Networks of excitatory and inhibitory neurons form the basic processing units in the cortex. Understanding the dynamical repertoire of such networks is therefore essential for understanding their input-output properties and identifying potential computational mechanisms in the brain.

One of the simplest models of a cortical network is a network of randomly connected units, the activity of each unit being represented by its instantaneous firing rate. A seminal study revealed that such networks can exhibit a transition from constant to strongly irregular activity when the coupling is increased [1]. Above the transition, the network displays a state in which the firing rates fluctuate strongly in time and across units, although the dynamics are fully deterministic and there are no external inputs. Such internally generated fluctuating activity is a signature of the chaotic nature of the dynamics [2–4], and the corresponding regime has been referred to as rate chaos. Recently, it has been proposed that this type of activity can serve as a substrate for complex computations [5]. Several works showed that the randomly connected rate network is able to learn complex temporal dynamics and input-output associations [6–8]. These computational properties may be related to the appearance of an exponential number of unstable fixed points at the transition [9], and to the fact that dynamics are slow and the signal-to-noise ratio maximal [10].

A natural question is whether actual cortical networks exhibit a dynamical regime analogous to rate chaos [19]. The classical network model analyzed in [1] and subsequent studies [6, 7, 11–15] contains several simplifying features that prevent a direct comparison with more biologically constrained models such as networks of spiking neurons. In particular, a major simplification is a high degree of symmetry in both input currents and firing rates. Indeed, in the classical model the synaptic strengths are symmetrically distributed around zero, and excitatory and inhibitory neurons are not segregated into different populations, thus violating Dale’s law. The current-to-rate activation function is furthermore symmetric around zero, so that the dynamics are symmetric under sign reversal. As a consequence, the mean activity in the network is always zero, and the transition to the fluctuating regime is characterized solely in terms of second order statistics.

To help bridge the gap between the classical model and more realistic spiking networks [18, 19], recent works have investigated fluctuating activity in rate networks that include additional biological constraints [16, 17, 19], such as segregated excitatory-and-inhibitory populations, positive firing rates and spiking noise [16]. In particular, two of those works [16, 17] extended to excitatory-inhibitory networks the dynamical mean field (DMF) theory used for the analysis of rate chaos in classical works [1]. In general excitatory-inhibitory networks, the DMF equations however proved difficult to solve, and these works focused instead mostly on the case of purely inhibitory networks. These works therefore left unexplained some phenomena observed in simulations of excitatory-inhibitory spiking and rate networks [19–21], in particular the observation that the onset of fluctuating activity is accompanied by a large elevation of mean firing rate [19], and the finding that fluctuating activity at strong coupling is highly sensitive to the upper bound [21].

Here we investigate the effects of excitation on fluctuating activity in inhibition-dominated excitatory-inhibitory networks [22–27]. To this end, we focus on a simplified network architecture in which excitatory and inhibitory neurons receive statistically identical inputs [18]. For that architecture, dynamical mean field equations can be solved. We find that in presence of excitation, the coupling between mean and the auto-correlation of the activity leads to a strong increase of mean firing rates in the fluctuating regime [19], a phenomenon that is much weaker in purely inhibitory networks. Moreover, as the coupling is increased, two different regimes of fluctuating activity appear: at intermediate coupling, the fluctuations are of moderate amplitude and stabilized by inhibition; at strong coupling, the fluctuations become very large, and are stabilized only by an upper bound on the activity, even if inhibition globally dominates. The second regime is highly robust to external or spiking noise, and appears also in more general network architectures. Finally we show that networks of spiking neurons exhibit signatures characteristic of these different regimes.

## Results

We consider a large, randomly connected network of excitatory and inhibitory rate units similar to previous studies [16, 17]. The network dynamics are given by:

$$\dot{x_{i}}\left(t\right)=-x_{i}\left(t\right)+\sum_{j=1}^{N}J_{ij}\phi \left(x_{j}\left(t\right)\right)+I$$ (1)

where *N* is the total number of units, *x*_*i*_ represents the total input current to unit *i*, and *J*_*ij*_ is the strength of synaptic inputs from unit *j* to unit *i*. In most of the results which follow, we will not include any external currents (*I* = 0). The function *ϕ*(*x*) is a monotonic, positively defined activation function that transforms input currents into output activity. For mathematical convenience, in most of the analysis we use a threshold-linear activation with an upper-bound *ϕ*_*max*_ (see Methods).

We focus on a sparse, two-population synaptic matrix identical to [18, 19]. We first study the simplest version in which all neurons receive the same number *C* ≪ *N* of incoming connections (respectively *C*_*E*_ = *fC* and *C*_*I*_ = (1 − *f*)*C* excitatory and inhibitory inputs). All the excitatory synapses have strength *J* and all inhibitory synapses have strength −*gJ*, but the precise pattern of connections is assigned randomly. For such connectivity, excitatory and inhibitory neurons are statistically equivalent as they receive statistically identical inputs. This situation greatly simplifies the mathematical analysis, and allows us to obtain results in a transparent manner. In a second step, we show that the obtained results extend to more general types of connectivity.

### Emergence of fluctuations in deterministic networks

#### Dynamical systems analysis

For a fixed, randomly chosen connectivity matrix, the network we consider is fully deterministic, and can therefore be examined in a first approach using standard dynamical system techniques [28]. Such an analysis has been performed in a number of previous studies (see e.g. [19, 23]), here we include it for completeness.

As the inputs to all units are statistically identical, the network admits a homogeneous fixed point in which the activity is constant in time and identical for all units, given by:

$$x_{0}=J\left(C_{E}-gC_{I}\right)\phi \left(x_{0}\right)+I.$$ (2)

The linear stability of this fixed point is determined by the eigenvalues of the matrix *S*_*ij*_ = *ϕ*′(*x*_0_)*J*_*ij*_. If the real parts of all eigenvalues are smaller than one, the fixed point is stable, otherwise it is linearly unstable.

For large networks, the eigenspectrum of *J*_*ij*_ consists of a part that is densely distributed in the complex plane over a circle of radius $J\sqrt{C_{E}+g^{2}C_{I}}$, and of a real outlier given by the effective balance of excitation and inhibition in the connectivity *J*(*C*_*E*_ − *gC*_*I*_) [29–31]. We focus here on an inhibition-dominated network corresponding to *g* > *C*_*E*_/*C*_*I*_. In this regime, the real outlier is always negative and the stability of the fixed point depends only on the densely distributed part of the eigenspectrum. The radius of the eigenspectrum disk, in particular, increases with the coupling *J*, and an instability occurs when the radius crosses unity. The critical coupling *J*_0_ is given by:

$$\phi^{\prime }\left(x_{0}\right)J_{0}\sqrt{C_{E}+g^{2}C_{I}}=1$$ (3)

where *x*_0_ depends implicitly on *J* through Eq (2) and the gain *ϕ*′(*x*) is in general finite and non-negative for all the values of *x*.

**Fig 1:**
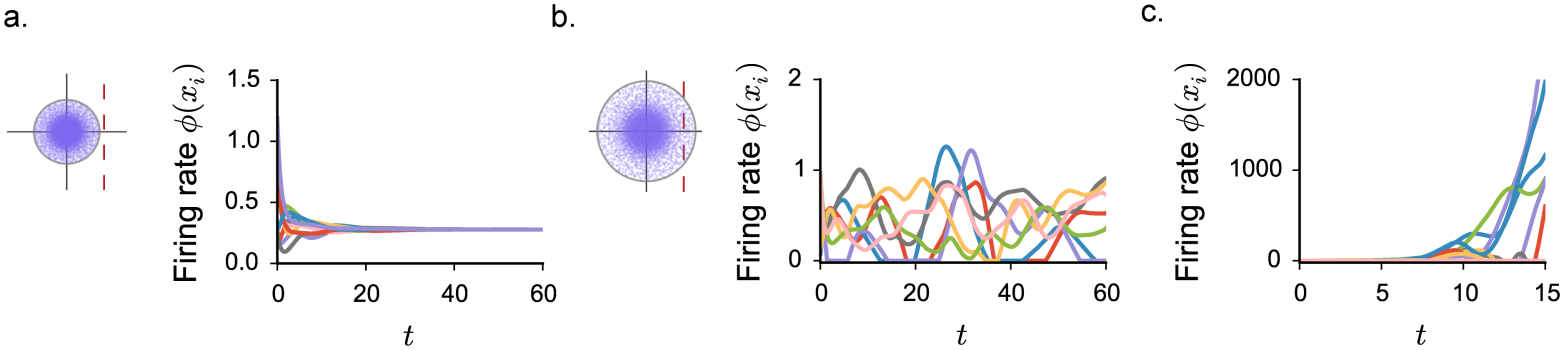
Dynamical regimes of an excitatory-inhibitory network of threshold-linear units as the coupling is increased. Numerical integration of the dynamics in Eq (1), firing rates of randomly chosen units. In the insets: complex eigenspectrum of the fixed point stability matrix, the red line corresponding to the stability bound. **a.** Weak coupling regime: the network activity converges to the homogeneous fixed point. **b.** Intermediate coupling regime: the activity displays stable fluctuations in time and across different units. **c.** Strong coupling regime: in absence of an upper bound, activity diverges. Choice of the parameters: *g* = 4.5, *C* = 100. *N* = 2000, no saturating upper bound: *ϕ*_*max*_ → ∞. In this and all other figures, all quantities are unitless (see Methods).

Numerical simulations confirm that, when *J* < *J*_0_, network activity settles into the homogeneous fixed point given by Eq (2) (Fig 1a). For *J* > *J*_0_, the fixed point is unstable, and the network exhibits ongoing dynamics in which the activities of different neurons fluctuate irregularly both in time and across units (Fig 1b). As the system is deterministic, these fluctuations are generated intrinsically in the network by strong feedback along unstable modes, which possess a random structure inherited from the random connectivity matrix.

#### Dynamical mean field description

The irregular, fluctuating activity regime cannot be easily analyzed with the tools of classical dynamical systems. Rather than attempting to describe single trajectories, we follow a different approach and focus on their statistics determined by averaging over time, instances of the connectivity matrix and initial conditions. To this end, we exploit mean field methods initially introduced for stochastic systems consisting of large numbers of units [32]. More specifically, we apply to our specific network architecture the dynamical mean field approach previously developed for similar deterministic networks [1, 2, 11, 16, 17].

Dynamical Mean Field (DMF) acts by replacing the fully deterministic interacting network by an equivalent stochastic system. As the interaction between units ∑_*j*_ *J*_*ij*_*ϕ*(*x*_*j*_) consists of a sum of a large number of terms, it can be replaced by a Gaussian stochastic process *η*_*i*_(*t*). Such a replacement provides an exact mathematical description under specific assumptions on the chaotic nature of the dynamics [33], and for particular limits of large network size *N* and number of connections *C*. Here we will treat it as an approximation, and we will assess the accuracy of this approximation by comparing the results with simulations performed for fixed *C* and *N* (see Methods for the limits of this approximation).

Replacing the interaction terms by Gaussian processes transforms the system into *N* identical Langevin-like equations:

$$\dot{x_{i}}\left(t\right)=-x_{i}\left(t\right)+\eta_{i}\left(t\right).$$ (4)

As *η*_*i*_(*t*) is a Gaussian noise, each trajectory *x*_*i*_(*t*) emerges thus as a Gaussian stochastic process, characterized by its first- and second-order moments. Within DMF, the mean and correlations of this stochastic process are determined self-consistently, by replacing averages over *η*_*i*_ with averages over time, instances of the connectivity matrix and initial conditions in the original system. In the limit of a large network, the stochastic processes corresponding to different units become uncorrelated. Moreover, in the specific network architecture considered here, all units are statistically equivalent, so that the network is effectively described by a single process. Note that in more general excitatory-inhibitory networks, a distinction needs to be made between different classes of neurons, and the DMF description becomes more complex [16, 17]. The details of the mean field analysis are provided in Methods.

The final outcome of DMF is a set of two equations for the first- and second-order statistics of the network activity. The equations are written in terms of the mean [*ϕ*] and autocorrelation *C*(*τ*) of the firing rate and the mean *μ* and mean-subtracted autocorrelation Δ(*τ*) of the input currents. The two sets of statistics provide an equivalent description of activity and have to respect self-consistency:

$$\left[\phi \right]=\int Dz\phi \left(\mu +\sqrt{\Delta_{0}}z\right)$$ (5)

$$C\left(\tau \right)=\int Dz{\left[\int Dy\phi (\mu +\sqrt{\Delta_{0}-\left|\Delta \left(\tau \right)\right|}y+\sqrt{\left|\Delta (\tau )\right|}z)\right]}^{2}$$ (6)

where

$$\mu =J\left(C_{E}-gC_{I}\right)\left[\phi \right]+I$$ (7)

$$C\left(\tau \right)=[\phi \left(x_{i}\left(t\right)\right)\phi \left(x_{i}\left(t+\tau \right)\right)]$$ (8)

$$\Delta \left(\tau \right)=\left[x_{i}\left(t\right)x_{i}\left(t+\tau \right)\right]-{\left[x_{i}\right]}^{2}.$$ (9)

In Eqs (5) and (6) we used the short-hand notation: $\int Dz=\int_{-\infty }^{+\infty }\frac{e^{-\frac{z^{2}}{2}}}{\sqrt{2\pi }}dz$, and Δ_0_ = Δ(*τ* = 0). Note that since all the units are statistically equivalent, [*ϕ*] and *C*(*τ*) are independent of the index *i*. The input current correlation function Δ(*τ*) moreover obeys an evolution equation in which the mean [*ϕ*] enters:

$$\ddot{\Delta }\left(\tau \right)=\Delta \left(\tau \right)-J^{2}\left(C_{E}+g^{2}C_{I}\right)\left\{C\left(\tau \right)-{\left[\phi \right]}^{2}\right\}.$$ (10)

The main difference here with respect to classical works [1] is that the first-order statistics are not trivial. In the classical case, the mean input *μ* is zero by construction, and the activation function *ϕ*(*x*) = tanh(*x*) is symmetric around zero, so that the mean firing rate [*ϕ*] in Eq (5) is zero. In our case, firing-rates are constrained to be positive, so that even in the case of perfect balance (*μ* = 0), the mean firing rate [*ϕ*] can in general be positive. We stress that as a consequence, the dynamics are described by coupled equations for the first- and second-order statistics rather than by second-order statistics alone (see also [16, 17]).

**Fig 2:**
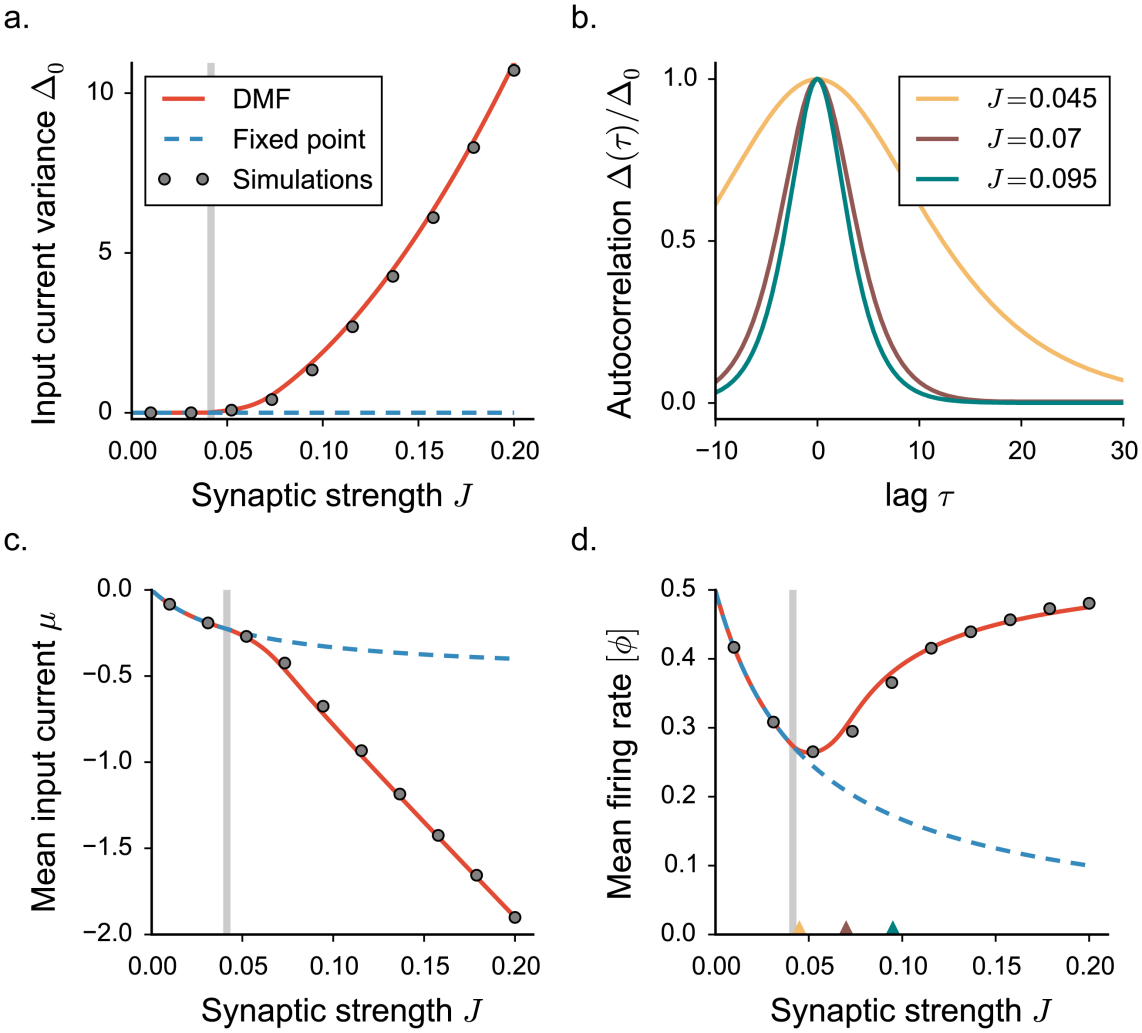
Statistical description of the network activity with a threshold-linear activation function. The dynamics mean field results are shown in full lines, numerical simulations as points. **a.** Input current variance as a function of the synaptic coupling *J*. Vertical grey lines indicate the critical value *J*_*C*_. Grey points show time and population averages performed on 4 realizations of simulated networks, *N* = 7000. **b.** Normalized auto-correlation function for increasing values of the synaptic coupling (indicated by colored triangles in panel **d**). **c-d.** First order statistics: mean input current and mean firing rate. Choice of the parameters: *g* = 5, *C* = 100, *ϕ*_*max*_ = 2.

Because all units are statistically equivalent, the DMF equations can be solved, and yield for each set of network parameters the mean-firing rate [*ϕ*], the mean input current *μ*, the current variance Δ_0_ and the current correlation function Δ(*τ*). Fig 2 shows a good match between theoretical predictions and numerically simulated activity. A more detailed analysis of finite size effects and limitations in DMF can be found in the Methods.

In agreement with the dynamical systems analysis, for low coupling values, DMF predicts a solution for which the variance Δ_0_ and the autocorrelation Δ(*τ*) of the fluctuations vanish at all times. Input currents set into a stationary and uniform value, corresponding to their mean *μ*. The predicted value of *μ* coincides with the fixed point *x*_0_, representing a low firing-rate background activity. As the coupling *J* is increased, the mean current becomes increasingly negative because inhibition dominates, and the mean firing rate decreases (Fig 2c and 2d).

For a critical coupling strength *J* = *J*_*C*_ (which coincides with *J*_0_, where the fixed point solution loses stability), DMF predicts the onset of a second solution with fluctuations of non-vanishing magnitude. Above *J*_*C*_, the variance of the activity grows smoothly from 0 (Fig 2a), and the auto-correlation Δ(*τ*) acquires a temporal structure, exponentially decaying to zero as *τ* → ∞. Close to the critical coupling, the dynamics exhibit a critical slowing down and the decay timescale diverges at *J*_*C*_, a behavior characteristic of a critical phase transition [1] (Fig 2b).

The onset of irregular, fluctuating activity is characterized by a transition of the second-order statistics from zero to a non-vanishing value. The appearance of fluctuations, however, directly affects also the first-order statistics. As the firing rates are constrained to be positive, large fluctuations induce deviations of the mean firing rate [*ϕ*] and the mean input current *μ* from their fixed point solutions. In particular, as *J* increases, larger and larger fluctuations in the current lead to an effective increase in the mean firing rate although the network is inhibition-dominated (Fig 2a, 2c and 2d). The increase in mean firing rate with synaptic coupling is therefore a signature of the onset of fluctuating activity in this class of excitatory-inhibitory networks.

In summary, intrinsically generated fluctuating activity in deterministic excitatory-inhibitory networks can be equivalently described by approximating the dynamics with a stationary stochastic process. Here we stressed that the mean and the autocorrelation of this process are strongly coupled and need to be determined self-consistently.

#### Two regimes of fluctuating activity

The mean field approach revealed that, above the critical coupling *J*_*C*_, the network generates fluctuating but stable, stationary activity. The dynamical systems analysis, however, showed that the dynamics of an equivalent linearized network are unstable and divergent for identical parameter values. The stability of the fluctuating activity is therefore necessarily due to the two non-linear constraints present in the system: the requirement that firing rates are bounded from below by 0 (i.e. positive), and the requirement that firing rates are limited by an upper bound *ϕ*_*max*_.

**Fig 3:**
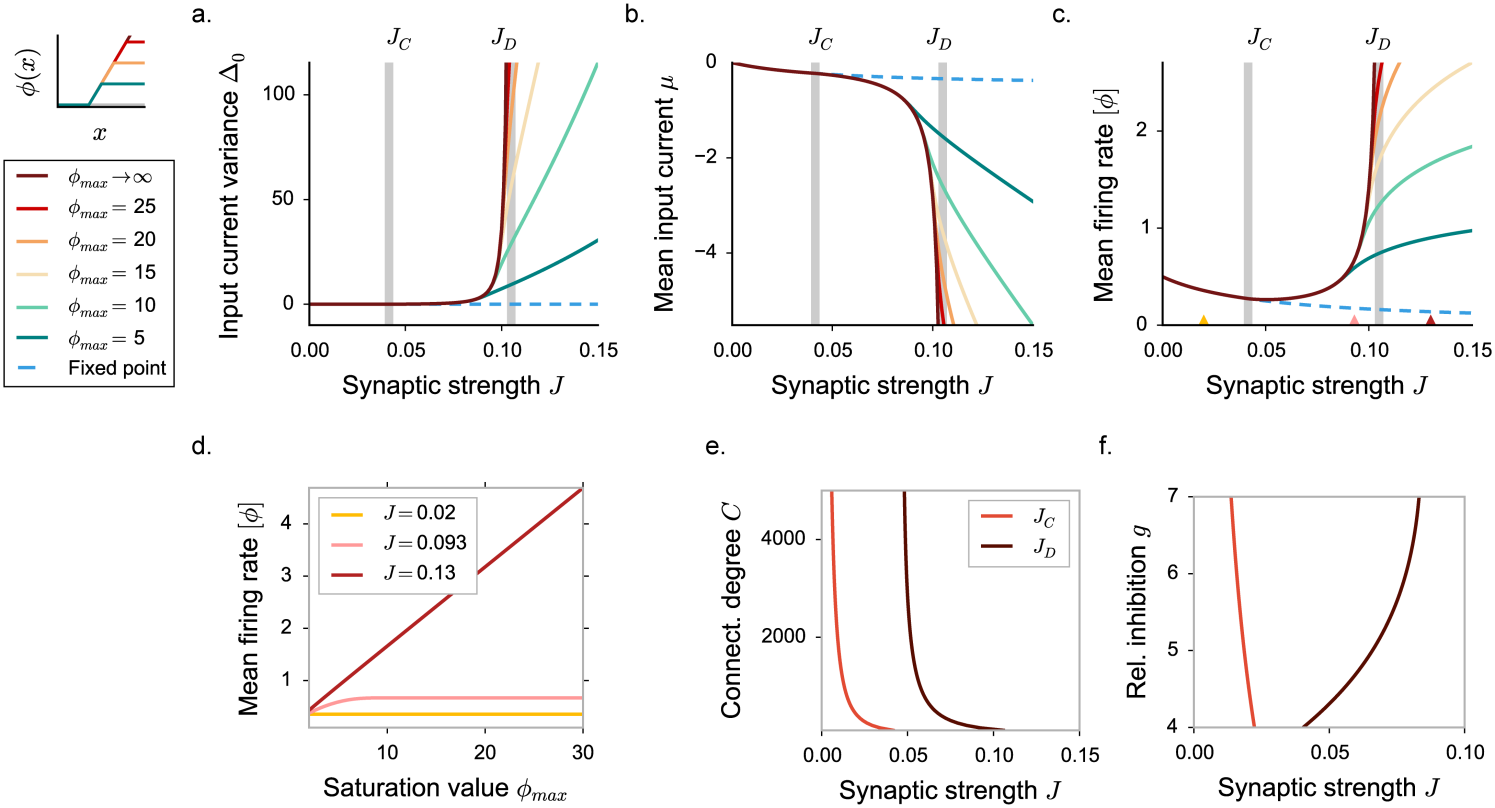
Appearance of three dynamical regimes in excitatory-inhibitory rate networks, dynamical mean field predictions. Threshold-linear activation function saturating at different values of the upper bound *ϕ*_*max*_. **a-b-c.** DMF characterization of the statistics for different values of the saturation value *ϕ*_*max*_. In **a**, input current variance, in **b**, input current mean, in **c**, mean firing rate. Vertical grey lines indicate the critical couplings *J*_*C*_ and *J*_*D*_. **d.** Mean firing rate dependence on the upper bound *ϕ*_*max*_, for three coupling values corresponding to the three different dynamical regimes (indicated by triangles in panel **c**). Dots show time and population averages performed on 4 realizations of simulated networks, *N* = 6000. Choice of the parameters: *g* = 5, *C* = 100. **e-f.** Phase diagram of the dynamics: dependence on the connectivity in-degree *C* and on the inhibition dominance parameter *g*. All other parameters are kept fixed.

In order to isolate the two contributions, we examined how the amplitude of fluctuating activity depends on the upper bound on firing rates *ϕ*_*max*_. Ultimately, we take this bound to infinity, leaving the activity unbounded. Solving the corresponding DMF equations revealed the presence of two qualitatively different regimes of fluctuating activity above *J*_*c*_ (Fig 3).

For intermediate coupling values, the magnitude of fluctuations and the mean firing rate depend only weakly on the upper bound *ϕ*_*max*_. In particular, for *ϕ*_*max*_ → ∞ the dynamics remain stable and bounded. The positive feedback that generates the linear instability is dominantly due to negative, inhibitory interactions multiplying positive firing rates in the linearized model. In this regime, the requirement that firing rates are positive, combined with dominant inhibition, is sufficient to stabilize this feedback and the fluctuating dynamics.

For larger coupling values, the dynamics depend strongly on the upper bound *ϕ*_*max*_. As *ϕ*_*max*_ is increased, the magnitude of fluctuations and the mean firing rate continuously increase and diverge for *ϕ*_*max*_ → ∞. For large coupling values, the fluctuating dynamics are therefore stabilized by the upper bound and become unstable in absence of saturation, even though inhibition is globally stronger than excitation.

Fig 3d summarizes the qualitative changes in the dependence on the upper bound *ϕ*_*max*_. In the fixed point regime, mean inputs are suppressed by inhibition, and they correspond to the low-gain region of *ϕ*(*x*), which is independent of *ϕ*_*max*_. Above *J*_*C*_, in the intermediate regime, the solution rapidly saturates to a limiting value. In the strong coupling regime, the mean firing rate, as well as the mean input *μ*, and its standard deviation $\sqrt{\Delta_{0}}$ grow linearly with the upper bound *ϕ*_*max*_. We observe that when *ϕ*_*max*_ is large, numerically simulated mean activity show larger deviations from the theoretically predicted value, because of larger finite size effects (for a more detailed discussion, see Methods).

The two regimes of fluctuating activity are characterized by different scalings of the first- and second-order statistics with the upper-bound *ϕ*_*max*_. In the absence of upper bound on the activity, i.e. in the limit *ϕ*_*max*_ → ∞, the two regimes are sharply separated by a second “critical” coupling *J*_*D*_: below *J*_*D*_, the network reaches a stable fluctuating steady-state and DMF admits a solution; above *J*_*D*_, the network has no stable steady-state, and DMF admits no solution. *J*_*D*_ corresponds to the value of the coupling for which the DMF solution diverges, and can be determined analytically (see Methods). For a fixed, finite value of the upper bound *ϕ*_*max*_, there is however no sign of transition as the coupling is increased past *J*_*D*_. Indeed, for a fixed *ϕ*_*max*_, the network reaches a stable fluctuating steady state on both sides of *J*_*D*_, and no qualitative difference is apparent between these two steady states. The difference appears only when the value of the upper bound *ϕ*_*max*_ is varied. *J*_*D*_ therefore separates two dynamical regimes in which the statistics of the activity scale differently with the upper-bound *ϕ*_*max*_, but for a fixed, finite *ϕ*_*max*_ it does not correspond to an instability. The second “critical” coupling *J*_*D*_ is therefore qualitatively different from the critical coupling *J*_*c*_, which is associated with an instability for any value of *ϕ*_*max*_.

The value of *J*_*D*_ depends both on the relative strength of inhibition *g*, and the total number of incoming connections *C*. Increasing either *g* or *C* increases the total variance of the interaction matrix *J*_*ij*_, shifting the instability of the homogeneous fixed point to lower couplings. The size of the intermediate fluctuating regime however depends only weakly on the number of incoming connections *C* (Fig 3e). In contrast, increasing the relative strength of inhibition diminishes the influence of the upper bound and enlarges the phase space region corresponding to the intermediate regime, where fluctuations are stabilized intrinsically by recurrent inhibition (Fig 3f). The second critical coupling *J*_*D*_ is in particular expected to increase with *g* and diverge for purely inhibitory networks. However, for very large relative inhibition, numerical simulations show strong deviations from DMF predictions, due to the breakdown of the Gaussian approximation which overestimates positive feedback (see Methods).

In summary, the two non-linearities induced by the two requirements that the firing rates are positive and bounded play asymmetrical roles in stabilizing fluctuating dynamics. In excitatory-inhibitory networks considered here, this asymmetry leads to two qualitatively different fluctuating regimes.

#### The effect of spiking noise

We next investigated whether the two different fluctuating regimes described above can be still observed when spiking noise is added to the dynamics. Following [15, 16], we added a Poisson spiking mechanism on the rate dynamics in Eq (1), and let the different units interact through spikes (see Methods). Within a mean field approach, interaction through spikes lead to an additive white noise term in the dynamics [15, 16]. To determine the effect of this additional term on the dynamics, we first treated it as external noise and systematically varied its amplitude as a free parameter.

**Fig 4:**
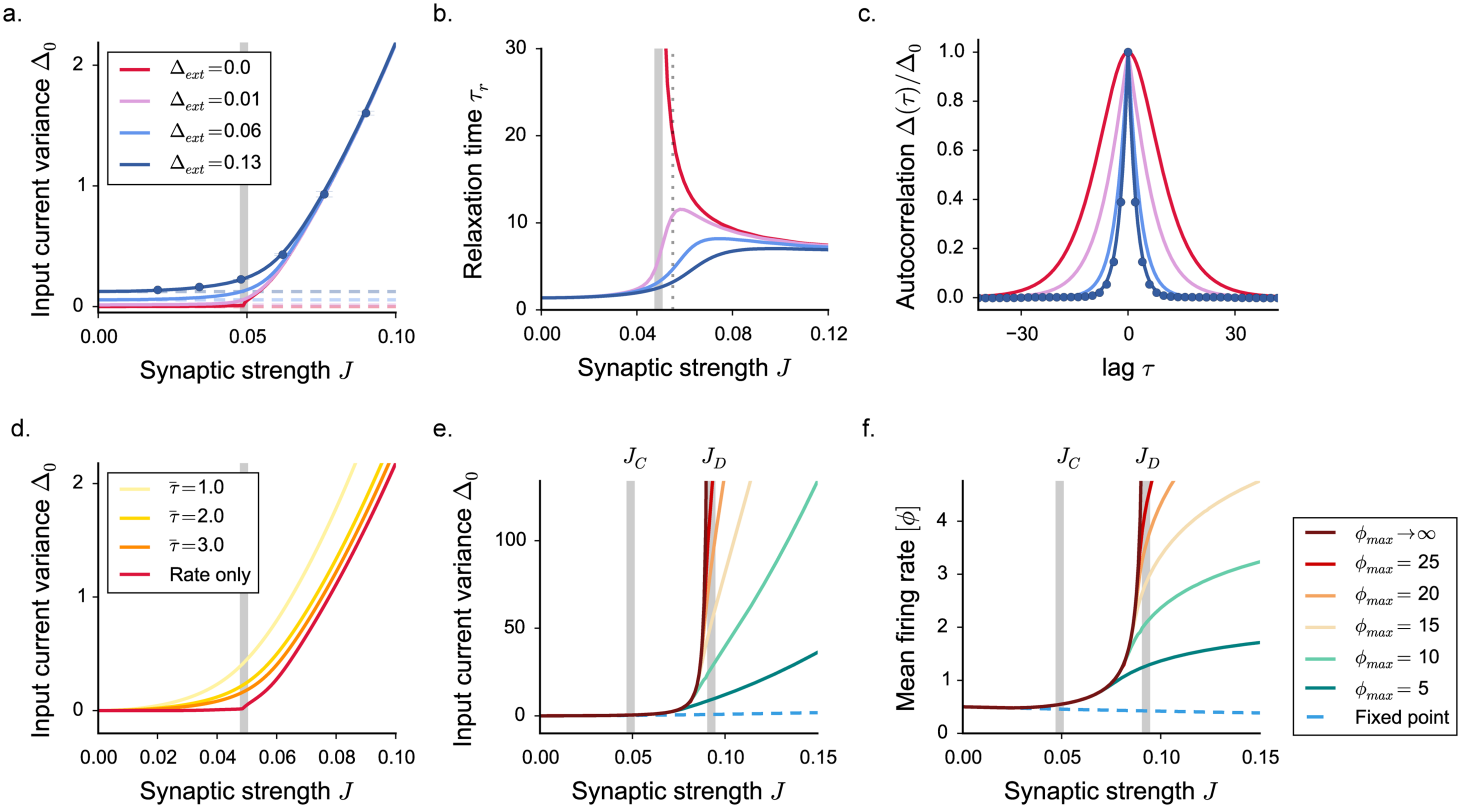
Statistical description of the activity in excitatory-inhibitory networks with external and spiking noise. The dynamical mean field results are shown in full lines, numerical simulations as points. **a.** Input current variance in presence of external noise, for increasing values of the noise amplitude (white noise, variance equal to 2Δ_*ext*_). Blue dots: results of numerical simulations for Δ_*ext*_ = 0.13, *N* = 7500, average of 4 realizations of the synaptic matrix. The grey vertical line shows the critical coupling *J*_*C*_ in the deterministic model. Dashed lines indicate the statistics of an effective fixed point, where the only variance is generated by the noise contribution Δ_*ext*_. The fixed point firing rate is computed as a Gaussian average, with the mean given by the fixed point *x*_0_ and the variance provided solely by the noise term. The deflection from the effective fixed point underlines an internal amplification of noise produced by network feedback. **b.** Fluctuations relaxation time, measured as the auto-correlation Δ(*τ*) full width at half maximum. **c.** Normalized auto-correlation for fixed *J* and different levels of noise. The corresponding coupling value is indicated by the dotted vertical gray line in panel **b**. **d.** Input variance in a network with spiking dynamics, where spikes are generated according to inhomogeneous Poisson processes. Increasing the time constant of rate dynamics $\bar{\tau }$ (see Eq (50) in Methods) decreases the amplitude of spiking noise. **e-f.** Appearance of the three dynamical regimes in a network with spiking noise: input current variance and mean firing rate for different saturation values *ϕ*_*max*_. Choice of the parameters: *g* = 4.1, *C* = 100.

The main effect of noise is to induce fluctuations in the activity for all values of network parameters (Fig 4a). As a result, in presence of noise, the sharp transition between constant and fluctuating activity is clearly lost as previously shown [15, 16]. The feedback mechanism that generates intrinsic fluctuations nevertheless still operates and strongly amplifies the fluctuations induced by external noise.

The DMF framework can be extended to include external noise and determine the additional variability generated by network feedback ([15, 16], see also Methods). When the coupling *J* is small, the temporal fluctuations in the activity are essentially generated by the filtering of external noise. Beyond the original transition at *J*_*C*_, instead, when the feedback fluctuations grow rapidly with synaptic coupling, the contribution of external noise becomes rapidly negligible with respect to the intrinsically-generated fluctuations (Fig 4a).

As shown in earlier studies [15, 16], a dramatic effect of introducing external noise is a strong reduction of the timescale of fluctuations close to *J*_*C*_. In absence of noise, just above the fixed point instability at *J*_*C*_, purely deterministic rate networks are characterized by the onset of infinitely slow fluctuations. These slow fluctuations are however of vanishingly small magnitude, and strongly sensitive to external noise. Any finite amount of external noise eliminates the diverging timescale. For weak external noise, a maximum in the timescale can be still seen close to *J*_*C*_, but it quickly disappears as the magnitude of noise is increased. For modest amounts of external noise, the timescale of the fluctuating dynamics becomes a monotonic function of synaptic coupling (Fig 4b).

While in presence of external noise there is therefore no formal critical phase transition, the dynamics still smoothly change from externally-generated fluctuations around a fixed point into intrinsically-generated, non-linear fluctuations. This change of regime is not necessarily reflected in the timescale of the dynamics, but can clearly be seen in the excess variance, and also in the first-order statistics such as the mean-firing rate, which again strongly increases with coupling. Moreover, we found that the existence of the second fluctuating regime is totally insensitive to noise: above the second critical coupling *J*_*D*_, the activity is only stabilized by the upper bound on the firing rates, and diverges in its absence. In that parameter region, intrinsically-generated fluctuations diverge, and the external noise contributes only a negligible amount.

We considered so far the effect of an external white noise of arbitrary amplitude. If that noise represents spiking interactions, its variance is however not a free parameter, but instead given by $J^{2}\left(C_{E}+g^{2}C_{I}\right)\left[\phi \right]/\bar{\tau }$. In particular, the amplitude of spiking noise increases both with the synaptic coupling and with the mean firing rate [*ϕ*], which itself depends on the coupling and fluctuations as pointed out above. As a result, the amplitude of the spiking noise dramatically increases in the fluctuating regime (Fig 4d). When *J* becomes close to the second critical coupling *J*_*D*_, the spiking noise however still contributes only weakly to the total variance (see in Methods), and the value of *J*_*D*_ is not affected by it (Fig 4e). The amplitude of spiking noise is also inversely proportional the timescale $\bar{\tau }$ of the dynamics (see Eq (50) in Methods). Slower dynamics tend to smooth out fluctuations due to spiking inputs (Fig 4d), reduce the amount of spiking and noise and therefore favor the appearance of slow fluctuations close to the critical coupling *J*_*c*_ [16].

In conclusion, the main findings reported above, the influence of intrinsically generated fluctuations on mean firing rate, and the existence of two different fluctuating regimes are still observed in presence of external or spike-generated noise. In particular, above the second transition, intrinsically generated fluctuations can be arbitrarily strong and therefore play the dominant role with respect to external or spiking noise.

#### Purely inhibitory networks

**Fig 5:**
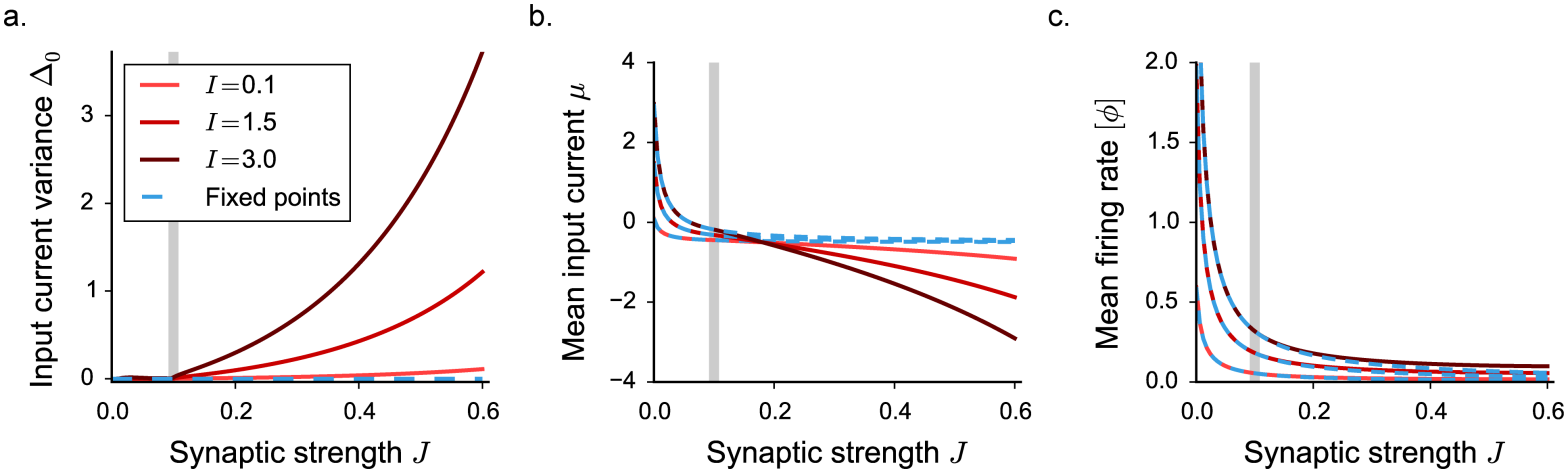
Statistical description of the activity in purely inhibitory networks. Results of the dynamical mean field theory (obtained through setting *C*_*E*_ = 0 and *g* = 1) for different values of the excitatory external current *I*. **a.** Input current variance, **b.** mean current and **c.** mean firing rate as a function of the synaptic coupling *J*. Vertical grey lines indicate the critical value *J*_*C*_.

To identify the specific role of excitation in the dynamics described above, we briefly consider here the case of networks consisting of a single inhibitory population. Purely inhibitory networks display a transition from a fixed point regime to chaotic fluctuations [16, 17]. The amplitude of fluctuations appears to be in general much smaller than in excitatory-inhibitory networks, but increases with the constant external current *I* (Fig 5a). In contrast to our findings for networks in which both excitation and inhibition are present, in purely inhibitory networks intrinsically generated fluctuations lead to a very weak increase in mean firing rates compared to the fixed point (Fig 5b and 5c). This effect can be understood by noting that within the dynamical mean field theory, the mean rate is given by (*μ* − *I*)/*J*(*C*_*E*_ − *gC*_*I*_) (Eq (7)). The term *C*_*E*_ − *gC*_*I*_ in the denominator determines the sensitivity of the mean firing rate to changes in mean input. This term is always negative as we are considering inhibition-dominated networks, but its absolute value is much smaller in presence of excitation, i.e. when excitation and inhibition approximately balance, compared to purely inhibitory networks. As the onset of intrinsically generated fluctuations modifies the value of the mean input with respect to its value in the fixed point solution (Figs 2c and 5b), this simplified argument explains why the mean firing rates in the inhibitory network are much less sensitive to fluctuations than in the excitatory-inhibitory case.

Moreover the second fluctuating regime found in EI networks does not appear in purely inhibitory networks. Indeed, the divergence of first- and second-order statistics that occurs in EI networks requires positive feedback that is absent in purely inhibitory networks. Note that for purely inhibitory, sparse networks, important deviations can exist at very large couplings between the dynamical mean field theory and simulations (see Methods for a more detailed discussion).

The two main findings reported above, the strong influence of intrinsically generated fluctuations on mean firing rate, and the existence of two different fluctuating regimes therefore critically rely on the presence of excitation in the network.

### Extensions to more general classes of networks

#### General excitatory-inhibitory (EI) networks

In the class of networks we investigated so far, excitatory and inhibitory units received statistically equivalent inputs. Under this assumption, the network dynamics are characterized by a single mean and variance for both excitatory and inhibitory populations, which considerably simplifies the mean field description. Here we relax this assumption and show that the properties of intrinsically generated fluctuations described so far do not critically depend on it.

We consider a more general class of networks, in which synaptic connections are arranged in a block matrix:

$$\text{J}=J\left(\frac{{\text{J}}_{\text{EE}}|}{{\text{J}}_{\text{IE}}|}\frac{{\text{J}}_{\text{EI}}}{{\text{J}}_{\text{II}}}\right)$$ (11)

where each block J_kk′_ is a sparse matrix, containing on each row *C*_*kk′*_ non-zero entries of value *j*_*kk′*_. The parameter *J* represents a global scaling on the intensity of the synaptic strength. For the sake of simplicity, we restrict ourselves to the following configuration: each row of *J* contains exactly *C*_*E*_ non-zero excitatory entries in the blocks of the excitatory column, and exactly *C*_*I*_ inhibitory entries in the inhibitory blocks. Non-zero elements are equal to *j*_*E*_ in J_EE_, to −*g*_*E*_ *j*_*E*_ in J_EI_, to *j*_*I*_ in J_IE_, and to −*g*_*I*_ *j*_*I*_ in J_II_. The previous case is recovered by setting *j*_*E*_ = *j*_*I*_ = 1 and *g*_*E*_ = *g*_*I*_.

The network admits a fixed point in which the activities are different for excitatory and inhibitory units, but homogeneous within the two populations. This fixed point is given by:

$$\left(\begin{array}{c} x_{0}^{E}\\ x_{0}^{I} \end{array}\right)=J\left(\begin{array}{c} j_{E}\left(C_{E}\phi \left(x_{0}^{E}\right)-g_{E}C_{I}\phi \left(x_{0}^{I}\right)\right)\\ j_{I}\left(C_{E}\phi \left(x_{0}^{E}\right)-g_{I}C_{I}\phi \left(x_{0}^{I}\right)\right) \end{array}\right)$$ (12)

where $x_{0}^{E}$ and $x_{0}^{I}$ are the fixed-point inputs to the two populations.

The linear stability of the fixed point is determined by the eigenvalues of the matrix:

$$\text{S}=J\left(\frac{\phi^{\prime }\left(x_{0}^{E}\right){\text{J}}_{\text{EE}}|}{\phi^{\prime }\left(x_{0}^{E}\right){\text{J}}_{\text{IE}}|}\frac{\phi^{\prime }\left(x_{0}^{I}\right){\text{J}}_{\text{EI}}}{\phi^{\prime }\left(x_{0}^{I}\right){\text{J}}_{\text{II}}.}\right)$$ (13)

The fixed point is stable if the real part of all the eigenvalues is smaller than one. As for simple, column-like EI matrices, the eigenspectrum of *S* is composed of a discrete and a densely distributed part, in which the bulk of the eigenvalues are distributed on a circle in the complex plane [12, 13, 34]. The discrete component consists instead of two eigenvalues, which in general can be complex, potentially inducing various kinds of fixed point instabilities (for the details, see Methods). As in the previous paragraphs, we consider a regime where both *g*_*E*_ and *g*_*I*_ are strong enough to dominate excitation, and the outlier eigenvalues have negative real part. In those conditions, the first instability to occur is the chaotic one, where the radius of the complex circle of the eigenspectrum crosses unity. This radius increases with the overall coupling *J*, defining a critical value *J*_*C*_ where the fixed point loses stability.

Dynamical mean field equations for the fluctuating regime above the instability are, in this general case, much harder to solve as they now involve two means and two auto-correlation functions, one for each populations [16, 17]. For that reason, we restrict ourselves to a slightly different dynamical system with discrete-time evolution:

$$x_{i}\left(t+1\right)=\sum_{j=1}^{N}J_{ij}\phi \left(x_{j}\left(t\right)\right).$$ (14)

**Fig 6:**
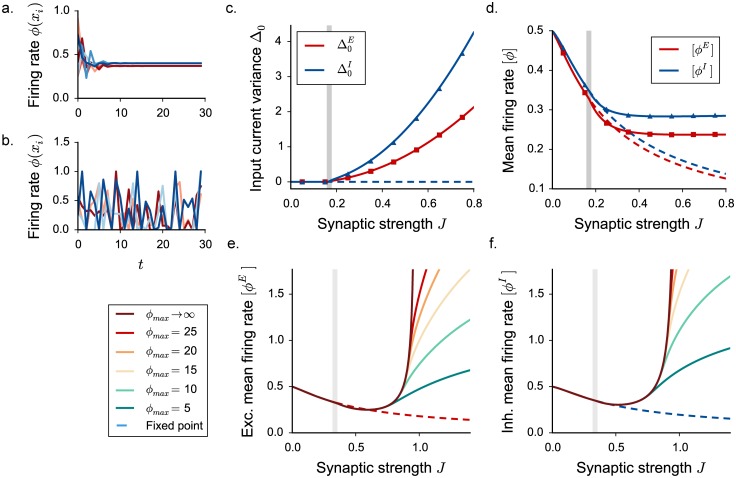
Fluctuating dynamics in more general networks where excitatory and inhibitory neurons are not statistically equivalent. Discrete-time rate evolution. **a-b.** Network discrete-time activity: numerical integration of the Eq (14), firing rates of randomly selected units. Excitatory neurons are plotted in the red scale, inhibitory ones in the blue one. *N* = 1000. In **a**, *J* < *J*_*C*_; in **b**, *J* > *J*_*C*_. **c-d.** Statistical characterization of network activity, respectively in terms of the input variance and the mean firing rate. Dynamical mean field results are shown in full lines. Dashed lines: fixed points. Dots: numerical simulations, *N* = 7500, average over 3 realizations. Vertical grey lines indicate the critical value *J*_*C*_. *ϕ*_*max*_ = 1. **e-f.** Mean firing rate for different values of the saturation *ϕ*_*max*_, in the excitatory and the inhibitory population. Choice of the parameters: *j*_*E*_ = 0.1, *j*_*I*_ = 1.5*j*_*E*_, *g*_*E*_ = 4.5, *g*_*I*_ = 4.2, *C* = 100.

Such a network corresponds to extremely fast dynamics with no current filtering (Fig 6a and 6b). Previous works [2–4, 10] have studied that class of models in case of synaptic matrices that lacked EI separation, and for activation functions that were symmetric. These works pointed out strong analogies with the dynamics emerging in continuous time [1]. Discrete-time dynamics can however induce a new, period-doubling bifurcation when inhibition is strong. We therefore restrict the analysis to a regime where inhibition is dominating but not excessively strong. Notice that in general, outside the range of parameters considered in this analysis, we expect generic EI networks to display a richer variety of dynamical regimes.

To begin with, we observe that the fixed-point (Eq (12)) and its stability conditions (Eq (13)) are identical for continuous and discrete dynamics. For discrete time, the DMF equations are however much simpler than for continuous dynamics, and can be easily fully solved even if the two populations are characterized now by different values of mean and variance.

Solving the DMF equations confirms that the transition to chaos in this class of models is characterized by the same qualitative features as before (Fig 6c and 6d). As the order parameter *J* is increased, the means and the variances of both the E and the I population display a transition from the fixed point solution to a fluctuating regime characterized by positive variance Δ_0_ and increasing mean firing rate. By smoothly increasing the upper bound of the saturation function *ϕ*_*max*_ as before, we find a second critical value *J*_*D*_ at which the firing activity of both populations diverge (Fig 6e and 6f). We conclude that the distinction in three regimes reported so far can be extended to discrete-time dynamics; in this simplified framework, our results extend to more general EI connectivity matrices.

#### Connectivity with stochastic in-degree

We now turn to networks in which the number of incoming connections is not fixed for all the neurons, but fluctuates stochastically around a mean value *C*. We consider a connectivity scheme in which each excitatory (resp. inhibitory) neuron makes a connection of strength *J* (resp. −*gJ*) with probability *C*/*N*.

In this class of networks, the number of incoming connections per neuron has a variance equal to the mean. As a consequence, in the stationary state, the total input strongly varies among units. In contrast to the case of a fixed in-degree, the network does not admit an homogeneous, but a heterogeneous fixed point in which different units reach different equilibrium values depending on the details of the connectivity.

The dynamical mean field approach can be extended to include the heterogeneity generated by the variable number of incoming connections [10, 16, 17]. As derived in Methods, the stationary distributions are now described by a mean and a static variance Δ_0_ that quantifies the static, quenched noise generated by variations in the total input among the units in the population. These two quantities obey:

$$\begin{array}{cc} & \mu =J\left(C_{E}-gC_{I}\right)\left[\phi \right]+I,\\ & \Delta_{0}=J^{2}\left(C_{E}+g^{2}C_{I}\right)\left[\phi^{2}\right]. \end{array}$$ (15)

**Fig 7:**
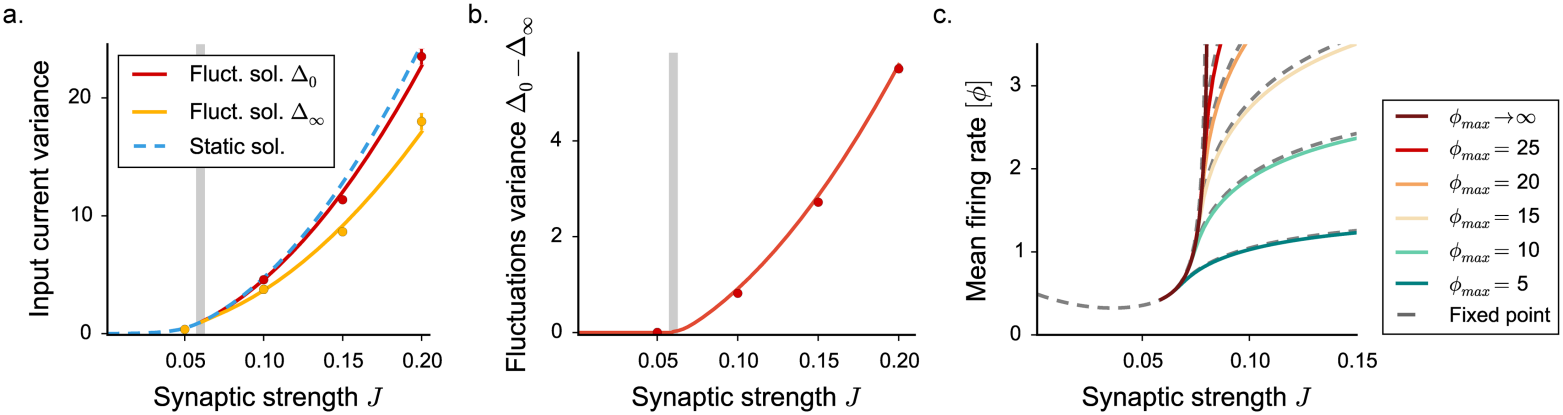
Mean field characterization of the activity in networks with stochastic in-degree. The dynamical mean field results are shown in full lines, numerical simulations as points. **(a)** Total input current variance Δ_0_. The heterogeneity in the connectivity induces an additional quenched variance Δ_∞_ (shown in dashed blue for the fixed point, and yellow for the fluctuating solution, where it corresponds to Δ_0_). Red (resp. yellow) points show time and population averages of Δ_0_ (resp. Δ_∞_) performed on 3 realizations of simulated networks, *N* = 6500. **(b)** Isolated contribution of temporal fluctuations to the variance. **(c)** Mean firing rate, for different values of the saturation *ϕ*_*max*_. Grey dashed lines indicate the stationary solution, becoming a thick colored line, corresponding to the chaotic phase, at *J*_*C*_. Choice of the parameters: *g* = 5, *C* = 100, *ϕ*_*max*_ = 2.

The stationary solution loses stability at a critical value *J* = *J*_*C*_. In the strong coupling regimes, DMF predicts the onset of a time-dependent solution with a decaying autocorrelation function, with initial condition Δ_0_ and asymptotic value Δ_∞_. The values of *μ*, Δ_0_ and Δ_∞_ are determined as solution of a system of three equations (see Eqs (79), (81) and (82) in Methods). In this regime, the effective amplitude of temporal fluctuations is given by the difference Δ_0_ − Δ_∞_ (Fig 7b). A non-zero value of Δ_∞_ reflects the variance of mean activity across the population: the the activity of different units fluctuates around different mean values because of the heterogeneity in the connectivity. Note moreover that because the static variance increases strongly with coupling (Fig 7a), the mean activity for the static solution increases with coupling, in contrast to the fixed in-degree case. In the fluctuating regime, as the additional temporal variance Δ_0_ − Δ_∞_ is weaker than the static variance Δ_∞_, temporal fluctuations do not lead to an increase in mean firing rate with respect to the static solution (Fig 7c), in contrast to our findings for the fixed in-degree case.

Fig 7c displays the dependence on the upper bound *ϕ*_*max*_ of the mean field solution. Above *J*_*C*_, an intermediate regime exists where the activity is stabilized by inhibition, and remains finite even in absence of upper bound. For couplings above a second critical coupling *J*_*D*_, the dynamics are stabilized only by the upper bound *ϕ*_*max*_. Networks with variable in-degree therefore show the same three dynamical regimes as networks with fixed degree.

### Comparing rate and integrate-and-fire networks

For excitatory-inhibitory networks of threshold-linear rate units, we have identified two different regimes of fluctuating activity. In this section, we show that networks of spiking, leaky integrate-and-fire (LIF) neurons display the signatures characteristic of these two regimes. To link threshold-linear rate networks to LIF networks, we first consider a modified rate model directly related to LIF networks [19], and then perform simulations of spiking LIF networks.

#### Rate networks with an LIF transfer function

We focus again on the fixed in-degree synaptic matrix in which the inputs to excitatory and inhibitory neurons are statistically equivalent, but consider a rate network in which the dynamics are now given by:

$$\dot{\phi_{i}}\left(t\right)=-\phi_{i}\left(t\right)+F\left(\mu_{i}\left(t\right),\sigma_{i}\left(t\right)\right)$$ (16)

where:

$$\begin{array}{cc} & \mu_{i}\left(t\right)=\mu_{0}+\tau_{m}\sum_{j}J_{ij}\phi_{j}\left(t\right)\\ & \sigma_{i}^{2}\left(t\right)=\tau_{m}\sum_{j}J_{ij}^{2}\phi_{j}\left(t\right). \end{array}$$ (17)

Here *ϕ*_*i*_ is the firing rate of unit *i*, *μ*_0_ is a constant external input, and *τ*_*m*_ = 20 ms is the membrane time constant. The function *F*(*μ*, *σ*) is the input-output function of a leaky integrate-and-fire neuron receiving a white-noise input of mean *μ* and variance *σ* [35]:

$$F\left(\mu ,\sigma^{2}\right)={\left[\tau_{rp}+2\tau_{m}\int_{\frac{V_{r}-\mu }{\sigma }}^{\frac{V_{th}-\mu }{\sigma }}\;due^{u^{2}}\int_{-\infty }^{u}\;d\nu e^{-\nu^{2}}\right]}^{-1}$$ (18)

where *V*_*th*_ and *V*_*r*_ are the threshold and reset potentials of the LIF neurons, and *τ*_*rp*_ is the refractory period.

The firing-rate model defined in Eq (16) is directly related to the mean field theory for networks of LIF neurons interacting through instantaneous synapses [18, 19, 36]. More specifically, the fixed point of the dynamics defined in Eq (16) is identical to the equilibrium firing rate in the classical asynchronous state of a network of LIF neurons with an identical connectivity as the rate model [18, 36]. Eq (16) can then be seen as simplified dynamics around this equilibrium point [37, 38]. A linear stability analysis of the fixed point for the rate model predicts an instability analogous to the one found in threshold-linear rate models. A comparison with a network of LIF neurons shows that this instability predicts a change in the dynamics in the corresponding spiking network, although there may be quantitative deviations in the precise location of the instability [19–21].

The dynamics of Eq (16) have been analytically investigated only up to the instability [19]. To investigate the dynamics above the instability, we set $x_{i}\left(t\right)=\sum_{j=1}^{N}J_{ij}\phi_{j}\left(t\right)$, and rewrite the dynamics in the more familiar form:

$$\dot{x_{i}}\left(t\right)=-x_{i}\left(t\right)+\sum_{j=1}^{N}J_{ij}F\left(\tau_{m}x_{j}\left(t\right),\sigma_{j}\left(t\right)\right).$$ (19)

The main novelty with respect to previously studied rate models is that the input-output transfer function *F* depends on the standard deviation *σ*_*j*_ of the input current to the unit *j*. A dependence on a time-varying *σ*_*j*_ is however difficult to include in the dynamical mean field approach. As a step forward, we fix *σ*_*j*_ to its average value independent of *j* and time, which corresponds to substituting all the firing rates with a constant effective value $\bar{\phi }$:

$$\sigma^{2}∼\tau_{m}\sum_{j}J_{ij}^{2}\bar{\phi }=\tau_{m}J^{2}\left(C_{E}+g^{2}C_{I}\right)\bar{\phi }.$$ (20)

With this substitution, we are back to a classical rate model with an LIF transfer function. Quantitatively the dynamics of that model are not identical to the model defined in Eq (16), but they can be studied using dynamical mean field theory. We therefore focus on qualitative features of the dynamics rather than quantitative comparisons between models.

**Fig 8:**
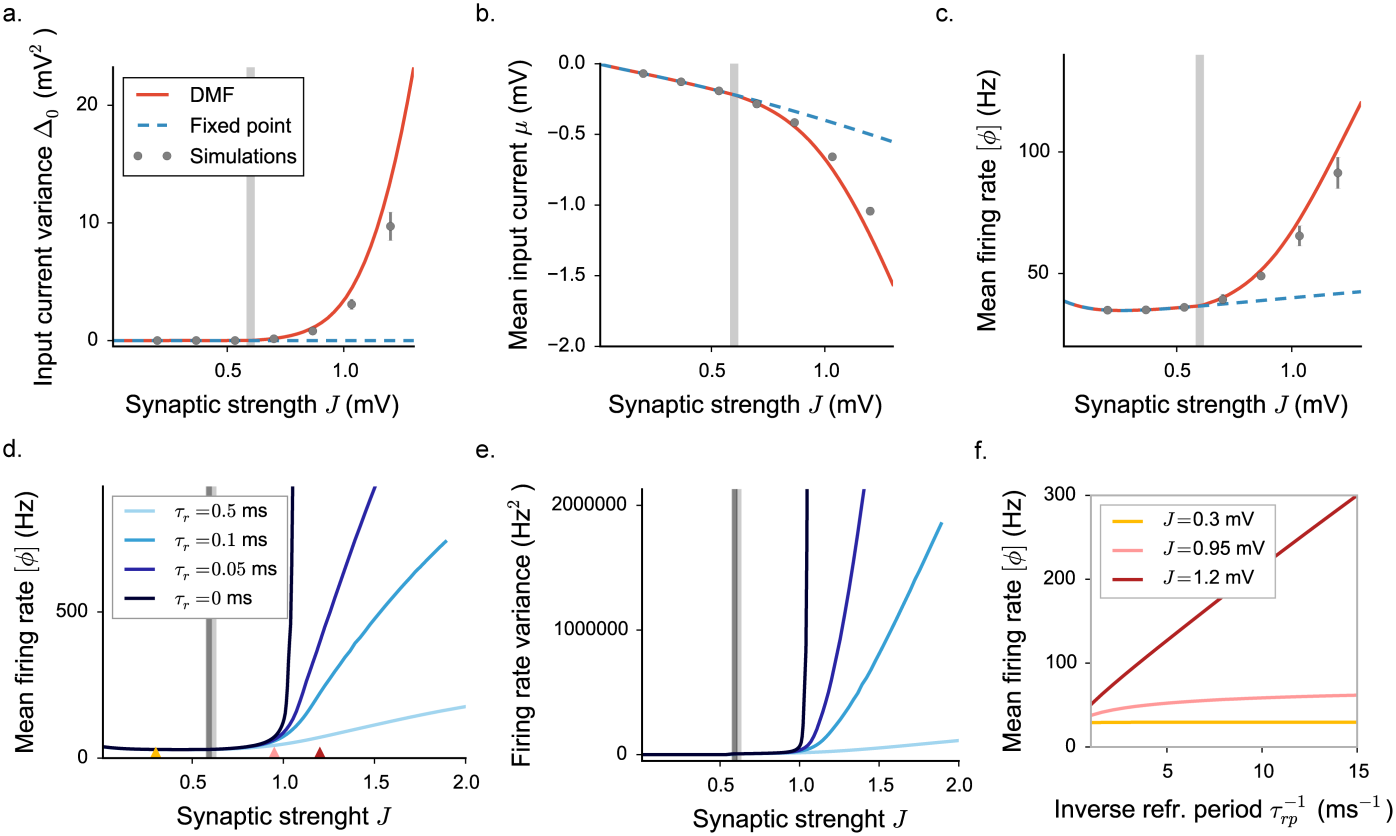
Dynamical mean field characterization of rate network activity with a LIF activation function, where we set $\sigma^{2}=\tau_{m}J^{2}\left(C_{E}+g^{2}C_{I}\right)\bar{\phi }$, $\bar{\phi }=20$ Hz. **a-b-c.** Statistical characterization for *τ*_*r*_ = 0.5 ms: input variance, mean input current and mean firing rate. Grey vertical lines indicate the position of the critical coupling. Choice of the parameters: *g* = 5, *C* = 100. **d-e.** Mean firing rate and rate standard deviation for different values of the refractory period, determining slightly different positions of the transition (grey lines). Choice of the parameters: *g* = 5, *C* = 100, *μ*_0_ = 24 mV. **f.** Mean firing rate dependence on the refractory period, the inverse of which determines the saturation value of the transfer function. The three values of the synaptic coupling, indicated by triangles in **c**, correspond to the three different regimes.

Solving the dynamical mean field equations shows that the dynamics in the rate model with and LIF transfer function are qualitatively similar to the threshold-linear rate model studied above. As the coupling strength *J* is increased above a critical value, the fixed point loses stability, and a fluctuating regime emerges. The amplitude of the fluctuations increases with coupling (Fig 8a), and induces an increase of the mean firing rate with respect to values predicted for the fixed point (Fig 8c).

In the LIF transfer function, the upper bound on the firing rate is given by the inverse of the refractory period. For that transfer function, changing the refractory period does not modify only the upper bound, but instead affects the full function. For different values of the refractory periods, the fixed point firing rate and the location of the instability therefore change, but these effects are very small for refractory periods below one millisecond.

Varying the refractory period reveals two different fluctuating regimes as found in threshold-linear rate models (Fig 8d, 8e and 8f). At intermediate couplings, the fluctuating dynamics depend weakly on the refractory period and remain bounded if the refractory period is set to zero. At strong couplings, the fluctuating dynamics are stabilized only by the presence of the upper bound, and diverge if the refractory period is set to zero. The main difference with the threshold-linear model is that the additional dependence on the coupling *J* induced by *σ* on the transfer function reduces the extent of the intermediate regime.

#### Spiking networks of leaky integrate-and-fire neurons

**Fig 9:**
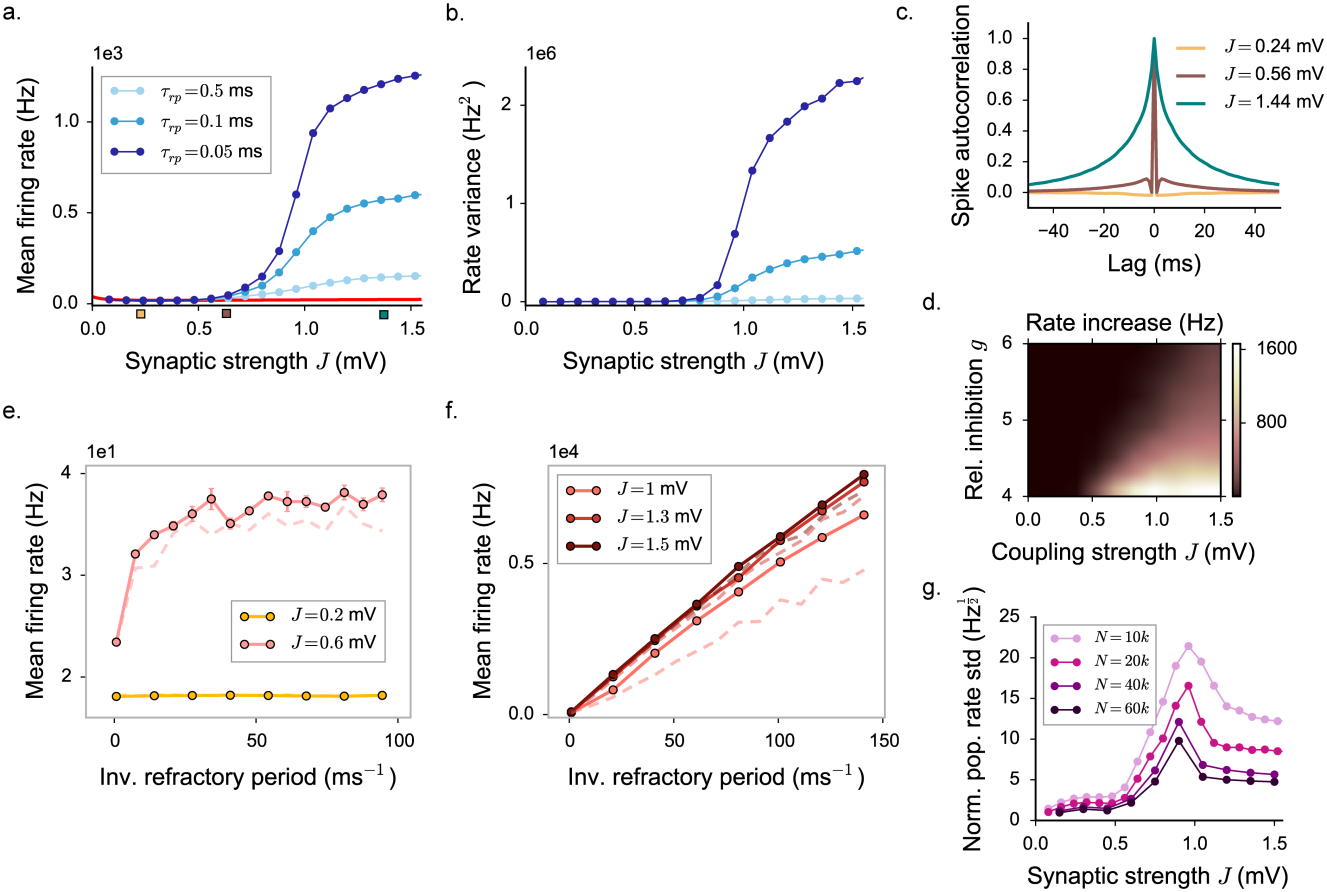
Statistical characterization of activity in a network of leaky integrate-and-fire neurons. **a.** Mean firing rate. Numerical simulations (*N* = 20000) are in good agreement with the LIF mean field prediction (red line) for low coupling values (*J* < 0.5). For high values of *J* (*J* > 0.8), mean firing rates diverge and becomes highly dependent on the refractory period. **b.** Firing rate variance, computed on instantaneous firing rates evaluated with a 50 ms Gaussian filter. **c.** Spike autocorrelation function, computed with 1 ms time bins, for three different values of the coupling *J* (*τ*_*rp*_ = 0.5). **d.** Increase in the mean firing rate as the refractory period is decreased from 0.5 to 0.1 ms, as a function of the synaptic coupling *J* and the inhibition strength *g*. As in the rate networks, the mean firing rate and its increase depend on the value of *g*. **e-f.** Direct dependence between the mean firing rate and refractory period. Panel **e** shows the low and intermediate coupling regime. Panel **f** shows the high coupling regime. Colored dots: simulated networks with *N* = 20000. Lighter dashed lines (when visible) show the result for *N* = 10000. **g.** Dependence on *J* and *N* of correlations and synchrony, quantified by the std of the population-averaged spiking rate, normalized by the square root of the mean firing rate (*τ*_*rp*_ = 0.05). Std is computed within a time bin of 1 ms. In all the panels, choice of the parameters: *g* = 5, *C* = 500, Δ = 1.1 ms, *μ*_0_ = 24 mV.

Having established the existence of two different regimes of fluctuating activity in rate networks with an LIF transfer function, we next consider spiking networks of LIF neurons. To compare the different regimes of activity in spiking networks with the regimes we found in rate networks, we performed direct numerical simulations of a spiking LIF network. We examined the effects of the coupling strength and refractory period on first- and second-order statistics (Fig 9a and 9b), i.e. the mean firing rate and the variance of the activity (computed on instantaneous firing rates evaluated with a 50 ms Gaussian filter).

For low couplings strengths, the mean firing-rate in the network is close to the value predicted for the fixed point of Eq (16), i.e. the equilibrium asynchronous state, and essentially independent of the refractory period. Similarly, the variance of the activity remains at low values independent of the refractory period. As the synaptic strength is increased, the mean firing rate deviates positively from the equilibrium value (Fig 9a), and the variance of the activity increases (Fig 9b). For intermediate and strong synaptic coupling, the values of first- and second-order activity statistics become dependent on the values of the refractory period.

Specifically, for intermediate values of the coupling, the mean-firing rate increases with decreasing refractory period, but saturates with decreasing refractory period (Fig 9e. This is similar to the behavior of the rate networks in the inhibition-stabilized fluctuating regime. For large values of the coupling, the mean-firing rate instead diverges linearly with the inverse of the refractory period (Fig 9f), a behavior analogous to rate networks in the second fluctuating regime in which the dynamics are only stabilized by the upper bound on the activity. The strength of the sensitivity to the refractory period depends on the inhibitory coupling: the stronger the relative inhibitory coupling, the weaker the sensitivity to the refractory period (Fig 9d).

The main qualitative signatures of the two fluctuating regimes found in networks of rate units are therefore also observed in networks of spiking LIF neurons. It should be however noted that the details of the dynamics are different in rate and LIF networks. In particular, the shape of auto-correlation functions is different, as LIF neurons display a richer temporal structure at low and intermediate coupling strengths. At strong coupling, the auto-correlation function resembles those of rate networks with spiking interactions (see Fig 4c), in particular it displays a characteristic cusp at zero time-lag. The simulated LIF networks show no sign of critical slowing down, as expected from the analysis of the effects of spiking noise on the activity.

Moreover, strong finite-size effects are present in the simulations. To quantify correlations among units and synchrony effects deriving from finite-size effects, we measure the standard deviation of the amplitude of fluctuations in the population-averaged activity, normalized by the square root of the mean firing rate (Fig 9g). Correlations and synchrony appear to be stronger for small values of the refractory period. The effect of correlations is furthermore weaker in the low and high coupling regimes, and it has a maximum for intermediate couplings. However, whatever the value of *J*, they decay as the system size is increased (for a more detailed characterization, see Methods).

In summary, for the range of values of the refractory period considered here, the activity in a network of spiking neurons is in qualitative agreement with predictions of the simple rate models analyzed in the previous sections. The rate model introduced in Eq (16) however does not provide exact quantitative predictions for the firing rate statistics above the instability. In particular, due to the numerical limitations in considering the limit *τ*_*rp*_ → 0, it is not possible to evaluate exactly through simulations the position of an equivalent critical value *J*_*D*_.

## Discussion

We investigated the fluctuating dynamics of sparsely connected rate networks with segregated excitatory and inhibitory subpopulations. We focused on a simplified network architecture, in which excitatory and inhibitory neurons receive statistically equivalent inputs, but differ in their output synaptic weights. In that case, the dynamical mean field equations that describe the dynamics can be fully analyzed.

Our central result is that in presence of excitation, two different regimes of fluctuating activity appear as coupling is increased. The distinction between these two regimes rests on whether the lower or the upper bound on activity stabilize network activity. At intermediate couplings, the fluctuating activity is stabilized by the lower bound that enforces positive firing rates, and remains finite even in absence of upper bound. For very strong coupling, the upper bound plays instead the dominant role, as in its absence fluctuations become unstable and the network shows run-away activity. This second fluctuating regimes is absent in purely inhibitory networks as it requires excitatory feedback.

We also showed that in presence of excitation, in networks with fixed in-degree, self-generated fluctuations strongly affect first order statistics such as the mean firing rate, which display important deviations from values predicted for the fixed point for identical coupling strengths. Such deviations of mean firing rates are therefore a signature of underlying fluctuations [19]. At strong coupling, in the second fluctuating regime, both the first and second order statistics monotonically increase with the upper bound.

We solved rigorously the DMF equations in simplified networks, where the in-degree is fixed and excitatory and inhibitory neurons are statistically equivalent. We showed however that the classification into three regimes extends to more general networks with statistically distinguishable populations and heterogeneous in-degrees. In particular, signatures of the two different fluctuating regimes are clearly apparent even when the network receives strong external noise. Finally these signatures are also seen in networks of integrate-and-fire neurons, which display qualitatively similar dynamical features.

### Relation to previous works

The transition from fixed point to fluctuating activity was first studied by Sompolinsky, Crisanti and Sommers [1]. In that classical work, the connectivity was Gaussian and the activation function symmetric around zero, so that the dynamics exhibited a sign-reversal symmetry. An important consequence of this symmetry is that the mean activity was always zero, and the transition was characterized solely in terms of second-order statistics, which were described through a dynamical mean field equation.

Recent studies have examined more general and biologically plausible networks [12, 13, 16, 17]. Two of those studies [16, 17] derived dynamical mean field (DMF) equations to networks with segregated excitatory and inhibitory populations, and asymmetric, positively defined transfer functions. The DMF equations are however challenging to solve in the general case of two distinct excitatory and inhibitory populations (see Methods). The two studies [16, 17] therefore analyzed in detail DMF solutions for purely inhibitory networks, and explored fluctuating activity in excitatory-inhibitory networks mainly through simulations.

In contrast to these recent works, here we exploited a simplified network architecture, in which DMF equations can be solved for excitatory-inhibitory networks. We found the presence of excitation qualitatively changes the nature of the dynamics, even though inhibition dominates. In purely inhibitory networks, fluctuations are weaker than in excitatory-inhibitory networks, and as a result only weakly affect first-order statistics.

In [16], the authors used transfer functions without upper bounds, and found that the chaotic state can undergo an instability in which the activity diverges. This instability is directly related to the transition between the two fluctuating regimes which we studied in detail for bounded transfer functions. Here we showed that these two dynamical regimes can in fact be distinguished only if the upper bound is varied: for a fixed upper bound, there is no sign of a transition. Moreover, we showed that excitation is required for the appearance of the second fluctuating regime, as this regime relies on positive feedback. For purely inhibitory networks, in which positive feedback is absent, simulations show that the second fluctuating regime does not occur, although it is predicted by dynamical mean field theory: indeed DMF relies on a Gaussian approximation which does not restrict the interactions to be strictly negative, and therefore artifactually introduces positive feedback at strong coupling.

The previous studies [16, 17] focused on networks with random in-degree or Gaussian coupling. In such networks, the quenched component of the coupling matrix leads to quenched heterogeneity in the stationary solution. In the present work, we instead mostly studied networks with fixed in-degree. We showed that in such a setting a homogeneous distribution is the stable solution, so that the quenched variability is not required for the transition to fluctuating activity.

### Synaptic timescales and rate fluctuations in networks of integrate-and-fire neurons

Under which conditions a regime analogous to rate chaos develops in networks of integrate-and-fire neurons has been a topic of intense debate [16, 17, 19–21]. Two different scenarios have been proposed: (i) rate chaos develops in networks of spiking neurons only in the limit of very slow synaptic or membrane time-constants [16, 17]; (ii) rate chaos can develop in generic excitatory-inhibitory networks, i.e. for arbitrarily fast synaptic time-constants [19]. The heart of the debate has been the nature of the signature of rate chaos in spiking networks.

The classical signature of the transition to rate chaos is *critical slowing-down*, i.e. the divergence of the timescale of rate fluctuations close to the transition [1, 20]. Importantly, this signature can be observed only if the coupling is very close to the critical value. Moreover, as shown in [15, 16], and reproduced here (Fig 4), spiking interactions induce noise in the dynamics, and critical slowing down is very sensitive to the amplitude of such noise. The amplitude of this spiking noise is moreover proportional to $1/\sqrt{\bar{\tau }}$, where $\bar{\tau }$ is the timescale of the rate model, usually interpreted as the slowest timescale in the system (either membrane or synaptic timescale). Critical-slowing down can therefore be observed only when the membrane or synaptic timescales are very slow and filter out the spiking noise [16, 17].

Here we have shown that for networks with EI connectivity and positive firing rates, a novel signature of fluctuating activity appears simply at the level of mean and variance of firing-rates, which become highly sensitive to the upper bound at strong coupling. In contrast to critical slowing-down, this signature of strongly fluctuating activity manifests itself in a large range of couplings above the critical value. A second difference with critical slowing down is that this signature of fluctuating activity is very robust to noise, and therefore independent of the timescale of the synapses or membrane time constant. Simulations of networks of integrate-and-fire neurons reveal such signatures of underlying fluctuating activity for arbitrarily fast synaptic time-constants, although there is no sharp transition in terms of critical slowing down.

The results presented here therefore reconcile the two proposed scenarios. A sharp phase-transition to fluctuating activity characterized by critical slowing down appears only in the limit of very slow synaptic or membrane time-constants. For arbitrarily fast synaptic time-constants, there is no sharp phase transition, but instead a smooth cross-over to strongly fluctuating activity that manifests itself at larger couplings through high sensitivity to the upper bound of the activity.

### Mean-field theories and rate-based descriptions of integrate-and-fire networks

The dynamical mean field theory used here to analyze rate networks should be contrasted with mean field theories developed for integrate-and-fire networks. Classical mean field theories for networks of integrate-and-fire neurons lead to a self-consistent firing rate description of the equilibrium asynchronous state [18, 36, 39], but this effective description is however not consistent at the level of the second order statistics. Mean field theories for IF neurons assume indeed that the input to each neuron consists of white noise, originating from Poisson spiking; however the firing of an integrate-and-fire neuron in response to white-noise inputs is in general not Poisson [40], so that the Poisson assumption is not self-consistent. In spite of this, mean field theory predicts well the first-order statistics over a large parameter range [41], but fails at strong coupling when the activity is strongly non-Poisson [19].

Extending mean field theory to determine analytically self-consistent second-order statistics is challenging for spiking networks. Several numerical approaches have been developed [44–46], but their range of convergence appears to be limited. A recent analysis of that type has suggested the existence of an instability driven by second-order statistics as the coupling is increased [46].

A simpler route to incorporate non-trivial second order statistics in the mean field description is to describe the different neurons as Poisson processes with rates that vary in time. One way to do this is to replace every neuron by a linear-nonlinear (LN) unit that transforms its inputs into an output firing rate, and previous works have shown that such an approximation can lead to remarkably accurate results [37, 38, 47, 48]. If one moreover approximates the linear filter in the LN unit by an exponential, this approach results in a mapping from a network of integrate-and-fire neurons to a network of rate units with identical connectivity [19]. Note that such an approximation is not quantitatively accurate for the leaky integrate-and-fire model with fast synaptic timescales—indeed the linear response of that model contains a very fast component ($1/\sqrt{t}$ divergence in the impulse response at short times, see [37]). A single timescale exponential however describes much better dynamics of other models, such as the exponential integrate-and-fire [37]. The accuracy of the mapping from integrate-and-fire to rate networks also depends on synaptic timescales which influence both the amplitude of synaptic noise and the transfer function itself [42]. It has been argued that the mapping becomes exact in the limit of infinitely long timescales [17, 43].

In this study, we have analyzed rate networks using dynamical mean field theory. This version of mean field theory is different from the one used for integrate-and-fire networks as it determines self-consistently and analytically not only the first-order statistics, but also the second-order statistics, i.e. the full auto-correlation function of neural activity. Note that this is similar in spirit to the approach developed for integrate-and-fire networks [44–46], except that integrate-and-fire neurons are replaced by simpler, analytically tractable rate units. Dynamical mean field theory reveals that at large coupling, network feedback strongly amplifies the fluctuations in the activity, which in turn lead to an increase in mean firing rates, as seen in networks of spiking neurons [19]. The rate-model moreover correctly predicts that for strong coupling, the activity is highly sensitive to the upper bound set by the refractory period, although the mean activity is well below saturation.

As pointed out above, the mapping from an integrate-and-fire to a rate network is based on a number of approximations and simplifications. The fluctuating state in the rate network therefore does not in general lead to a quantitatively correct description of the activity in a network of integrate-and-fire neurons. However, the rate model does capture the existence of a fundamental instability, which amplifies fluctuations through network feedback.

## Methods

### Rate network model

We investigate the dynamics of a rate network given by:

$$\dot{x_{i}}\left(t\right)=-x_{i}\left(t\right)+\sum_{j=1}^{N}J_{ij}\phi \left(x_{j}\left(t\right)\right)+I$$ (21)

where the index *i* runs over the units of the network. Each variable *x*_*i*_ is interpreted as the total input current to neuron *i*, and *ϕ*(*x*) is a monotonic, positively defined activation function, transforming currents into output firing rates. *I* is a common external input and *J*_*ij*_ a random synaptic matrix. We have rescaled time to set the time constant to unity. All quantities are therefore taken to be unitless.

We consider a two-population (excitatory and inhibitory), sparsely connected network. All the excitatory synapses have strength *J*, while all inhibitory synapses have strength −*gJ*, the parameter *g* playing the role of the relative amount of inhibition over excitation. In the simplest model we consider, each neuron receives exactly *C* incoming connections, with 1 ≪ *C* ≪ *N* [18]. A fraction *f* of inputs are excitatory (*C*_*E*_ = *fC*), the remaining are inhibitory (*C*_*I*_ = (1 − *f*)*C*). We set *f* = 0.8.

For the sake of simplicity, in most of the applications we restrict ourself to the case of a threshold-linear activation function with an offset *γ*. For practical purposes, we take:

$$\phi \left(x\right)=\left\{\begin{array}{cc} 0 & x<-\gamma \\ \gamma +x & -\gamma \leq x\leq \phi_{max}-\gamma \\ \phi_{max} & x>\phi_{max}-\gamma \end{array}\right.$$ (22)

where *ϕ*_*max*_ plays the role of the saturation value. In the following, we set *γ* = 0.5. In most of the applications, if not explicitly indicated, we consider networks with no external input, and set *I* = 0.

### Mean field theory derivation

Here we derive in detail the Dynamical Mean Field (DMF) equations for the simplest excitatory-inhibitory network where the number of incoming connections *C* is identical for all units. For networks of large size, mean field theory provides a simple effective description of the network activity. More specifically, here we consider the limit of large *N* while *C* (and synaptic strengths) are held fixed [18, 39]. The derivation provided here follows the same steps as in [1, 11], but takes into account non-vanishing first moments.

The dynamics of the network depend on the specific realization of the random connectivity matrix. The evolution of the network can therefore be seen as a random process, which can be characterized by its moments, obtained by averaging over realizations of the connectivity matrix. The dynamics can be described either by the moments of the synaptic currents *x*_*i*_, or by moments of the firing rates *ϕ*(*x*_*i*_). The two sets of moments are coupled, and DMF theory exploits a Gaussian approximation to derive a closed set of equations for the first- and second-order moments. This closed set of equations can then be solved self-consistently.

More specifically, DMF theory acts by replacing the fully deterministic coupling term ∑_*j*_ *J*_*ij*_*ϕ*(*x*_*j*_) + *I* in Eq (21) by an equivalent Gaussian stochastic process *η*_*i*_. The effective mean field dynamics are therefore given by:

$$\dot{x_{i}}\left(t\right)=-x_{i}\left(t\right)+\eta_{i}\left(t\right)$$ (23)

where the distribution of *η*_*i*_ should effectively mimic the statistics of the original system in Eq (21).

To be able to compute the moments of the synaptic currents *x*_*i*_ and firing rates *ϕ*(*x*_*i*_), the first step is to compute self-consistently the first and second order moments of the effective noise *η*_*i*_. For this, averages over units, initial conditions, time and synaptic matrix instances (that we will indicate with 〈〉) are substituted with averages over the distribution of the stochastic process (that we will indicate with []). For the mean, we get:

$$\begin{array}{cc} & \left[\eta_{i}\left(t\right)\right]=\left\langle \sum_{j=1}^{N}J_{ij}\phi \left(x_{j}\right)+I\right\rangle =\sum_{j_{E}=1}^{C_{E}}J\left\langle \phi \left(x_{j_{E}}\right)\right\rangle -g\sum_{j_{I}=1}^{C_{I}}J\left\langle \phi \left(x_{j_{I}}\right)\right\rangle +I\\ & =J\left(C_{E}-gC_{I}\right)\left\langle \phi \right\rangle +I \end{array}$$ (24)

where the indices *j*_*E*_ and *j*_*I*_ run over the excitatory and the inhibitory units pre-synaptic to unit *i*.

Following previous works [1, 3], here we assume that, for large *N*, *J*_*ij*_ and *ϕ*(*x*_*j*_) behave independently. Moreover, we assume that the mean values of *x* and *ϕ* reach stationary values for *t* → ∞, such that [*η*_*i*_(*t*)] = [*η*_*i*_].

Under the same hypothesis, the second moment [*η*_*i*_(*t*)*η*_*j*_(*t* + *τ*)] is given by:

$$\left[\eta_{i}\left(t\right)\eta_{j}\left(t+\tau \right)\right]=\left\langle \sum_{k=1}^{N}J_{ik}\phi \left(x_{k}\left(t\right)\right)\sum_{l=1}^{N}J_{jl}\phi \left(x_{l}\left(t+\tau \right)\right)\right\rangle +2IJ\left(C_{E}-gC_{I}\right)\left\langle \phi \right\rangle +I^{2}.$$ (25)

In order to evaluate the first term in the r.h.s., we differentiate two cases: first, we take *i* = *j*, yielding the noise auto-correlation. We assume that in the thermodynamic limit, where the Langevin equations in Eq (23) decouple, different units behave independently: 〈*ϕ*(*x*_*k*_)*ϕ*(*x*_*l*_)〉 = 〈*ϕ*(*x*_*k*_)〉〈*ϕ*(*x*_*l*_)〉 if *k* ≠ *l*. We will verify this assumption self-consistently by showing that, in the same limit, [*η*_*i*_(*t*)*η*_*j*_(*t* + *τ*)] = 0 when *i* ≠ *j*.

The sum over *k* (*l*) can be split into a sum over *k*_*E*_ and *k*_*I*_ (*l*_*E*_ and *l*_*I*_) by segregating the contributions from the two populations. We thus get:

$$\begin{array}{cc} & \left\langle \sum_{k=1}^{N}J_{ik}\phi \left(x_{k}\left(t\right)\right)\sum_{l=1}^{N}J_{il}\phi \left(x_{l}\left(t+\tau \right)\right)\right\rangle =\left\langle \sum_{k_{E}=1}^{N_{E}}J_{ik_{E}}\phi \left(x_{k_{E}}\left(t\right)\right)\sum_{l_{E}=1}^{N_{E}}J_{il_{E}}\phi \left(x_{l_{E}}\left(t+\tau \right)\right)\right\rangle \\ & +\left\langle \sum_{k_{I}=1}^{N_{I}}J_{ik_{I}}\phi \left(x_{k_{I}}\left(t\right)\right)\sum_{l_{I}=1}^{N_{I}}J_{il_{I}}\phi \left(x_{l_{I}}\left(t+\tau \right)\right)\right\rangle +2\left\langle \sum_{k_{E}=1}^{N_{E}}J_{ik_{E}}\phi \left(x_{k_{E}}\left(t\right)\right)\sum_{l_{I}=1}^{N_{I}}J_{il_{I}}\phi \left(x_{l_{I}}\left(t+\tau \right)\right)\right\rangle . \end{array}$$ (26)

We focus on the first term of the sum (same considerations hold for the second two), and we differentiate contributions from *k*_*E*_ = *l*_*E*_ and *k*_*E*_ ≠ *l*_*E*_. Setting *k*_*E*_ = *l*_*E*_ returns a contribution equal to *C*_*E*_ *J*^2^〈*ϕ*^2^〉. In the sum with *k*_*E*_ ≠ *l*_*E*_, as *C* is fixed, we obtain exactly *C*_*E*_(*C*_*E*_ − 1) contributions of value *J*^2^〈*ϕ*〉^2^. This gives, for the two populations:

$$\begin{array}{cc} & \left\langle \sum_{k=1}^{N}J_{ik}\phi \left(x_{k}\left(t\right)\right)\sum_{l=1}^{N}J_{il}\phi \left(x_{l}\left(t+\tau \right)\right)\right\rangle =C_{E}J^{2}\left\langle \phi \left(x_{i}\left(t\right)\right)\phi \left(x_{i}\left(t+\tau \right)\right)\right\rangle +C_{E}\left(C_{E}-1\right)J^{2}{\left\langle \phi \right\rangle }^{2}\\ & -2C_{E}C_{I}gJ^{2}{\left\langle \phi \right\rangle }^{2}+C_{I}g^{2}J^{2}\left\langle \phi \left(x_{i}\left(t\right)\right)\phi \left(x_{i}\left(t+\tau \right)\right)\right\rangle +C_{I}\left(C_{I}-1\right)g^{2}J^{2}{\left\langle \phi \right\rangle }^{2}\\ & =J^{2}\left(C_{E}+g^{2}C_{I}\right)\left\langle \phi \left(x_{i}\left(t\right)\right)\phi \left(x_{i}\left(t+\tau \right)\right)\right\rangle +J^{2}{\left(C_{E}-gC_{I}\right)}^{2}{\left\langle \phi \right\rangle }^{2}-J^{2}\left(C_{E}+g^{2}C_{I}\right){\left\langle \phi \right\rangle }^{2}. \end{array}$$ (27)

By defining the rate auto-correlation function *C*(*τ*) = 〈*ϕ*(*x*_*i*_(*t*))*ϕ*(*x*_*i*_(*t* + *τ*))〉, we finally get:

$$\left[\eta_{i}\left(t\right)\eta_{i}\left(t+\tau \right)\right]-{\left[\eta_{i}\right]}^{2}=J^{2}\left(C_{E}+g^{2}C_{I}\right)\left\{C\left(\tau \right)-{\left\langle \phi \right\rangle }^{2}\right\}.$$ (28)

When *i* ≠ *j*, we instead obtain:

$$\begin{array}{cc} & \left\langle \sum_{k=1}^{N}J_{ik}\phi \left(x_{k}\left(t\right)\right)\sum_{l=1}^{N}J_{jl}\phi \left(x_{l}\left(t+\tau \right)\right)\right\rangle =C_{E}^{2}J^{2}{\left\langle \phi \right\rangle }^{2}+pC_{E}J^{2}\left\{C\left(\tau \right)-{\left\langle \phi \right\rangle }^{2}\right\}+C_{I}^{2}g^{2}J^{2}{\left\langle \phi \right\rangle }^{2}\\ & +pC_{I}g^{2}J^{2}\left\{C\left(\tau \right)-{\left\langle \phi \right\rangle }^{2}\right\}-2C_{E}C_{I}gJ^{2}{\left\langle \phi \right\rangle }^{2}. \end{array}$$ (29)

The constant *p* corresponds to the probability that, given that *k* is a pre-synaptic afferent of neuron *i*, the same neuron is connected also to neuron *j*. Because of sparsity, we expect this value to be small. More precisely, since *N* is assumed to be large, we can approximate the probability *p* with *C*/*N*. We eventually find:

$$\left[\eta_{i}\left(t\right)\eta_{j}\left(t+\tau \right)\right]-\left[\eta_{i}\right]\left[\eta_{j}\right]=pJ^{2}\left(C_{E}+g^{2}C_{I}\right)\left\{C\left(\tau \right)-{\left\langle \phi \right\rangle }^{2}\right\}\to 0$$ (30)

because *p* → 0 when *N* → ∞.

Once the statistics of the effective stochastic term *η*_*i*_ are known, we can describe the input current *x* in terms of its mean *μ* = [*x*_*i*_] and its mean-subtracted correlation function Δ(*τ*) = [*x*_*i*_(*t*)*x*_*i*_(*t* + *τ*)] − [*x*_*i*_]^2^. The mean field current *x*_*i*_(*t*) emerging from the stochastic process in Eq (23) behaves as a time-correlated Gaussian variable. First we observe that, asymptotically, its mean value *μ* coincides with the mean of the noise term *η*_*i*_:

$$\mu =J\left(C_{E}-gC_{I}\right)\left[\phi \right]+I$$ (31)

By differentiating twice Δ(*τ*) with respect to *τ* and using eqs (23) and (28), we moreover get a second-order differential equation for the auto-correlation evolution:

$$\ddot{\Delta }\left(\tau \right)=\Delta \left(\tau \right)-J^{2}\left(C_{E}+g^{2}C_{I}\right)\left\{C\left(\tau \right)-{\left\langle \phi \right\rangle }^{2}\right\}.$$ (32)

By explicitly constructing *x*(*t*) and *x*(*t* + *τ*) in terms of unit Gaussian variables, we self-consistently rewrite the firing rate statistics [*ϕ*] and *C*(*τ*), as integrals over the Gaussian distributions:

$$\begin{array}{cc} & \left[\phi \right]=\int Dz\phi \left(\mu +\sqrt{\Delta_{0}}z\right)\\ & C\left(\tau \right)=\int Dz{\left[\int Dy\phi (\mu +\sqrt{\Delta_{0}-\Delta \left(\tau \right)}y+\sqrt{\Delta (\tau )}z)\right]}^{2} \end{array}$$ (33)

where we used the short-hand notation: $\int Dz=\int_{-\infty }^{+\infty }\frac{e^{-\frac{z^{2}}{2}}}{\sqrt{2\pi }}dz$. For reasons which will become clearly soon, we can focus on positive values of the auto-correlation Δ. We moreover defined Δ_0_ = Δ(*τ* = 0).

Following [1], Eq (32) can be seen as analogous to the equation of motion of a classical particle in a one-dimensional potential:

$$\ddot{\Delta }=-\frac{\partial V(\Delta ,\Delta_{0})}{\partial \Delta }.$$ (34)

The potential *V*(Δ, Δ_0_) can be derived by integrating the right-hand side of Eq (32) over Δ and using Eq (33). This yields

$$V\left(\Delta ,\Delta_{0}\right)=-\frac{\Delta^{2}}{2}+J^{2}\left(C_{E}+g^{2}C_{I}\right)\left\{\int Dz{\left[\int Dy\Phi (\mu +\sqrt{\Delta_{0}-\Delta }y+\sqrt{\Delta }z)\right]}^{2}-\Delta {\left[\phi \right]}^{2}\right\}$$ (35)

where $\Phi \left(x\right)=\int_{-\infty }^{x}dz\phi \left(z\right)$.

In absence of external noise, the initial condition to be satisfied is $\dot{\Delta }\left(\tau =0\right)=0$, which implies null kinetic energy for *τ* = 0. A second condition is given by: Δ_0_ > |Δ(*τ*)| ∀*τ*. The solution Δ(*τ*) depends on the initial value Δ_0_, and it is governed by the energy conservation law:

$$V\left(\Delta \left(\tau =0\right),\Delta_{0}\right)=V\left(\Delta \left(\tau =\infty \right),\Delta_{0}\right)+\frac{1}{2}\dot{\Delta }{\left(\tau =\infty \right)}^{2}.$$ (36)

The stationary points and the qualitative features of the Δ(*τ*) trajectory depend then on the shape of the potential *V*. We notice that the derivative of the potential in Δ = 0 is always 0, suggesting a possible equilibrium point where the current distribution is concentrated in its mean value *μ*. Note that the existence of the stationary point in 0 stems from the −Δ[*ϕ*]^2^ term in the potential, which comes from taking the connectivity degree *C* fixed for each unit in the network (for a comparison with the equations obtained for random in-degree networks, see below).

**Fig 10:**
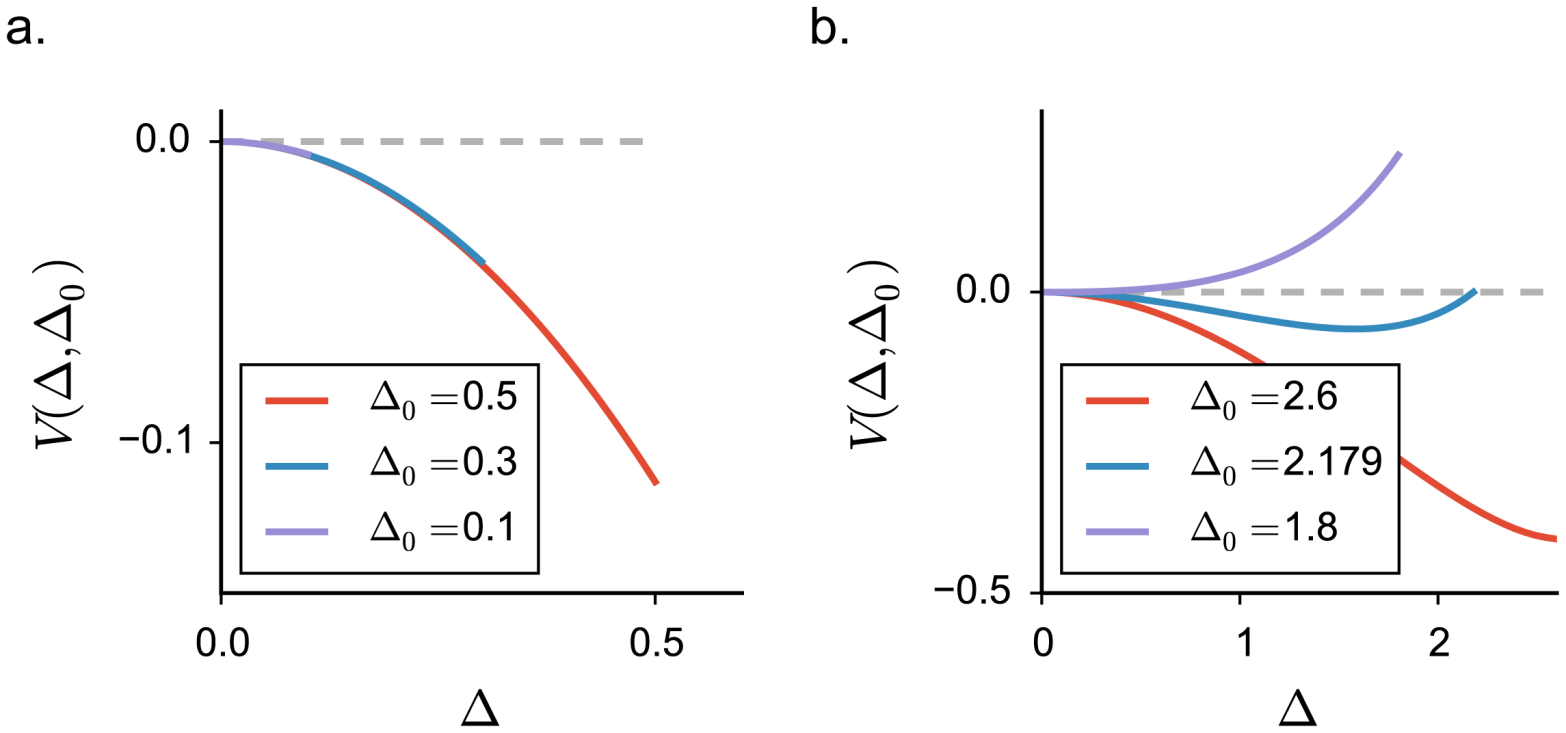
Dynamical mean field potential *V*(Δ, Δ_0_) for different values of the parameter Δ_0_; fixed *μ*. The activation function is chosen to be threshold-linear. **a.** *J* < *J*_*C*_: the potential is always concave. **b.** *J* > *J*_*C*_: the shape of the potential strongly depends on the value of Δ_0_.

When the first moment *μ* is determined self-consistently, the shape of *V* depends on the values of *J* and Δ_0_ (Fig 10a and 10b). In particular, a critical value *J*_*C*_ exists such that:

- when *J* < *J*_*C*_, the potential has the shape of a concave parabola centered in Δ = 0 (Fig 10a). The only physical bounded solution is then Δ = Δ_0_ = 0;
- when *J* > *J*_*C*_, the potential admits different qualitative configurations and an infinite number of different Δ(*τ*) trajectories. In general, the motion in the potential will be oscillatory (Fig 10b).

However, in the strong coupling regime, a particular solution exists, for which Δ(*τ*) decays to 0 as *τ* → ∞. In this final state, there is no kinetic energy left. A monotonically decaying auto-correlation function is the only stable solution emerging from numerical simulations.

For this particular class of solutions, (eq (36)) reads:

$$V\left(\Delta_{0},\Delta_{0}\right)=V\left(0,\Delta_{0}\right).$$ (37)

More explicitly:

$$\begin{array}{cc} \frac{\Delta_{0}^{2}}{2}=J^{2}\left(C_{E}+g^{2}C_{I}\right) & \left\{\int Dz\Phi^{2}\left(\mu +\sqrt{\Delta_{0}}z\right)-{\left(\int Dz\Phi (\mu +\sqrt{\Delta_{0}}z)\right)}^{2}\right.\\ & \left.-\Delta_{0}{\left(\int Dz\phi (\mu +\sqrt{\Delta_{0}}z)\right)}^{2}\right\} \end{array}$$ (38)

which gives an equation for *μ* and Δ_0_ to be solved together with the equation for the mean:

$$\mu =J\left(C_{E}-gC_{I}\right)\int Dz\phi \left(\mu +\sqrt{\Delta_{0}}z\right)+I$$ (39)

In a more compact form, we can reduce the system of equations to:

$$\begin{array}{cc} \mu = & J\left(C_{E}-gC_{I}\right)\left[\phi \right]+I\\ \frac{\Delta_{0}^{2}}{2}= & J^{2}\left(C_{E}+g^{2}C_{I}\right)\left\{\left[\Phi^{2}\right]-{\left[\Phi \right]}^{2}-\Delta_{0}{\left[\phi \right]}^{2}\right\}. \end{array}$$ (40)

Once *μ* and Δ_0_ are computed, their value can be injected into eq (32) to get the time course of the auto-correlation function.

Not surprisingly, the results above rely on the assumption of sparsity in the connectivity: *C* ≪ *N*. Classic DMF theory, indeed, requires synaptic entry *J*_*ij*_ to be independent one from each other. Fixing the number of non-zero connections for each unit is imposing a strong dependence among the entries in each row of the synaptic matrix. Nevertheless, we expect this dependence to become very weak when *N* → ∞, and we find that DMF can still predict correctly the system behavior, keeping however a trace of the network homogeneity through the term −[*ϕ*]^2^ in Eq (32). Fixing the degree *C* sets to zero the asymptotic value of the auto-correlation function, and results in a perfect self-averaging and homogeneity of activity statistics in the population.

To conclude, we note that finding the DMF solution for an excitatory-inhibitory network reduces here to solving a system of two-equations. A large simplification in the problem comes here from considering networks where excitatory and inhibitory units receive statistically equivalent inputs. DMF theory models indeed the statistical distribution of the input currents inside each network unit. For this reason, it does not include any element deriving from the segregation of the excitatory and the inhibitory populations in a two-columns connectivity structure. In consequence, for identical sets of parameters, we expect the same DMF equations to hold in more generic networks, where each neuron receive *C*_*E*_ excitatory and *C*_*I*_ inhibitory inputs, but can make excitatory or inhibitory output connections. We checked the validity of this observation (see later in Methods).

In a more general case, where excitation and inhibition are characterized as distinguishable populations with their own statistics, solving the DMF equations becomes computationally costly. The main complication comes from the absence of any equivalent classical motion in a potential. For that reason, previous studies have focused mostly on the case of purely inhibitory populations [16, 17].

#### Second critical coupling *J*_*D*_

While the DMF equations can be derived for a generic activation function *ϕ*(*x*), here we focus, for mathematical convenience, on the simple case of the threshold-linear activation function in Eq (22). From now on, for simplicity, we will set *I* = 0. For each value of the synaptic strength *J*, the system in Eq (40) allows to compute the first- and second-order statistics of the network activity. As shown in *Results*, DMF revels the existence of two different fluctuating regimes above the critical coupling *J*_*C*_, governed by the two different non-linear constraint in the dynamics: the positivity and the saturation of firing rates.

Here we study the behavior of the DMF solution close to the second critical coupling *J*_*D*_, in the case of a non-saturating activation function where *ϕ*_*max*_ → ∞. When *J* approaches *J*_*D*_, Δ_0_ → ∞, while *μ* → −∞ (Fig 3).

Led by dimensionality arguments, we assume that, close to the divergence point, the ratio $k=\mu /\sqrt{\Delta_{0}}$ is constant. With a threshold-linear transfer function, it is possible to compute analytically the three Gaussian integrals implicit in Eq (40) and to provide an explicit analytic form of the DMF equations. The equation for the mean translates into:

$$\mu =J\left(C_{E}-gC_{I}\right)\left[\phi \right]=J\left(C_{E}-gC_{I}\right)\left\{\left(\frac{1}{2}+\mu \right)\left(\frac{1}{2}-g\left(x_{a}\right)\right)+\sqrt{\frac{\Delta_{0}}{2\pi }}e^{-\frac{1}{2}x_{a}^{2}}\right\}.$$ (41)

where $x_{a}=\frac{1}{\sqrt{\Delta_{0}}}(-\frac{1}{2}-\mu )∼-k$ and where we have defined: $g\left(x\right)=\frac{1}{2}\mathrm{erf}\left(x/\sqrt{2}\right)$. When *J* → *J*_*D*_, by keeping only the leading order in $\sqrt{\Delta_{0}}$, we find $\mu =\hat{k}\sqrt{\Delta_{0}}$ with:

$$\hat{k}=\frac{J\left(C_{E}-gC_{I}\right)\frac{e^{-\frac{k^{2}}{2}}}{\sqrt{2\pi }}}{1-J\left(C_{E}-gC_{I}\right)\left(\frac{1}{2}+G\left(k\right)\right)}.$$ (42)

By imposing $k=\hat{k}$, one can determine self-consistently the value of *k* for each value of *J*. We introduce $\mu =k\sqrt{\Delta_{0}}$ into the second equation for Δ_0_. By keeping only the leading order in Δ_0_, we find:

$$\begin{array}{cc} & \sqrt{\Delta_{0}}=f\left(k\right)\\ f & \left(k\right)=\frac{J^{2}\left(C_{E}+g^{2}C_{I}\right)T\left(k\right)}{\frac{1}{2}-J^{2}\left(C_{E}+g^{2}C_{I}\right)S\left(k\right)} \end{array}$$ (43)

with:

$$\begin{array}{cc} S(k)= & \frac{1}{4}k^{4}\left[\frac{1}{2}+g\left(k\right)\right]+\frac{1}{4}k^{3}\frac{e^{-\frac{k^{2}}{2}}}{\sqrt{2\pi }}+k^{2}\left[\frac{3}{2}\left(\frac{1}{2}+g\left(k\right)\right)-{\left(\frac{1}{2}+g\left(k\right)\right)}^{2}\right]\\ & +k\left[\frac{5}{4}\frac{e^{-\frac{k^{2}}{2}}}{\sqrt{2\pi }}-2\left(\frac{1}{2}+g\left(k\right)\right)\frac{e^{-\frac{k^{2}}{2}}}{\sqrt{2\pi }}\right]+\frac{3}{4}\left(\frac{1}{2}+g\left(k\right)\right)-{\left(\frac{e^{-\frac{k^{2}}{2}}}{\sqrt{2\pi }}\right)}^{2}\\ & -{\left\{\left(\frac{1}{2}k^{2}+\frac{1}{2}\right)\left[\frac{1}{2}+g\left(k\right)\right]+\frac{1}{2}k\frac{e^{-\frac{k^{2}}{2}}}{\sqrt{2\pi }}\right\}}^{2} \end{array}$$ (44)

In order to obtain a solution Δ_0_, from Eq (43) we require the function *f*(*k*) to be positive. We observe that *f* diverges when its denominator crosses zero. Here *f*(*k*) changes sign, becoming negative. We use this condition to determine *J*_*D*_:

$$J_{D}^{2}\left(C_{E}+g^{2}C_{I}\right)S\left(k\left(J_{D}\right)\right)=\frac{1}{2}$$ (45)

In presence of external noise of variance 2Δ_*ext*_, the equation for Δ_0_ is perturbed by an additive term proportional to $\Delta_{ext}^{2}$ (see below in Methods). Since we treat the noise variance as a constant, this additional term does not contribute to the divergence in the leading order in Δ_0_ (namely, $\Delta_{0}^{3/2}$, $\Delta_{0}^{2}$), and the presence of noise does not influence the value of *J*_*D*_.

Similarly, when we add white noise to mimic the noise introduced by Poisson spikes, we find the extra term to be proportional to [*ϕ*]^2^, which is of the same order of Δ_0_. As a consequence, again, it does not perturb the equation for Δ_0_ to the leading orders.

#### Mean field theory in presence of noise

In order to investigate the effect of an external noisy input on the dynamical regimes, we introduced an additive, white noise term in Eq (21). The network dynamics in this case read:

$$\dot{x_{i}}\left(t\right)=-x_{i}\left(t\right)+\sum_{j=1}^{N}J_{ij}\phi \left(x_{j}\left(t\right)\right)+\xi_{i}\left(t\right)$$ (46)

with 〈*ξ*_*i*_(*t*)〉 = 0 and 〈*ξ*_*i*_(*t*)*ξ*_*j*_(*t* + *τ*)〉 = 2Δ_*ext*_ *δ*_*ij*_*δ*(*τ*).

As above, we replace the forcing term ∑_*j*_ *J*_*ij*_*ϕ*(*x*_*j*_) + *ξ*_*i*_ by an effective noise *η*_*i*_. By following the same steps as before we find:

$$\begin{array}{cc} & \left[\eta_{i}\left(t\right)\right]=J\left(C_{E}-gC_{I}\right)\left\langle \phi \right\rangle \\ & \left[\eta_{i}\left(t\right)\eta_{i}\left(t+\tau \right)\right]-{\left[\eta_{i}\right]}^{2}=\delta_{ij}\left[J^{2}\left(C_{E}+g^{2}C_{I}\right)\left\{C\left(\tau \right)-{\left\langle \phi \right\rangle }^{2}\right\}+2\Delta_{ext}\delta \left(\tau \right)\right] \end{array}$$ (47)

which translates into:

$$\ddot{\Delta }\left(\tau \right)=\Delta \left(\tau \right)-J^{2}\left(C_{E}+g^{2}C_{I}\right)\left\{C\left(\tau \right)-{\left[\phi \right]}^{2}\right\}+2\Delta_{ext}\delta \left(\tau \right)$$ (48)

We conclude that the external noise acts on the auto-correlation function by modifying its initial condition into: $\dot{\Delta }\left(0^{+}\right)=-\dot{\Delta }\left(0^{-}\right)=-\Delta_{ext}$. In terms of the analogy with the 1D motion, the presence of noise translates into an additive kinetic term in *τ* = 0, which one has to take into account while writing down the energy balance:

$$V\left(\Delta_{0},\Delta_{0}\right)+\frac{1}{2}\dot{\Delta }{\left(0\right)}^{2}=V\left(0,\Delta_{0}\right)$$ (49)

to be solved again together with the equation for the mean *μ*. The potential *V*(Δ, Δ_0_), in contrast, remains unperturbed. The main effect of including a kinetic term at *τ* = 0 consists in allowing a variance Δ_0_ ≠ 0 also in the low coupling regime, where the potential has the usual shape as in Fig 10a.

From a mean field perspective, white noise can be studied as a proxy for the effect induced by spikes on the rate dynamics. In order to better quantify this effect, following [16], we add a spiking mechanism on the rate dynamics in Eq (1). Spikes are emitted according to independent inhomogeneous Poisson processes of rate *ϕ*(*x*_*j*_(*t*)), which obeys:

$$\bar{\tau }\dot{x}\left(t\right)=-x\left(t\right)+\sum_{j=1}^{N}J_{ij}\chi_{j}\left(t\right)$$ (50)

and *χ*_*j*_(*t*) is the spike train emitted by neuron *j*: $\chi_{j}\left(t\right)=\sum_{k}\delta \left(t-t_{j}^{k}\right)$.

This simple spiking mechanism can be again incorporated into a DMF description. Here, following [16], we show that the resulting equations correspond to an usual rate model with additive white noise, whose variance is given by $J^{2}\left(C_{E}+g^{2}C_{I}\right)\left[\phi \right]/\bar{\tau }$. The mean field forcing noise is in this case *η*_*i*_(*t*) = ∑_*j*_ *J*_*ij*_*χ*_*j*_(*t*). By separating *J*_*ij*_ into the sum of its mean and a zero-mean term, we get that the usual equation for the first order statistics holds:

$$\left[\eta_{i}\right]=J\left(C_{E}-gC_{I}\right)\left[\phi \right]$$ (51)

In order to compute the noise auto-correlation, we separate *η*_*i*_ into a rate and a zero-mean spikes contribution: $\eta_{i}=\eta_{i}^{r}+\eta_{i}^{sp}$, where $\eta_{i}^{r}=\sum_{j}J_{ij}\phi \left(x_{j}\right)$ and $\eta_{i}^{sp}=\sum_{j}J_{ij}\left\{\chi_{j}-\phi \left(x_{j}\right)\right\}$. The auto-correlation of the rate component returns the usual contribution:

$$\left[\left(\eta_{i}^{r}\left(t\right)-\left[\eta_{i}^{r}\right]\right)\left(\eta_{j}^{r}\left(t+\tau \right)-\left[\eta_{j}^{r}\right]\right)\right]=\delta_{ij}J^{2}\left(C_{E}+g^{2}C_{I}\right)\left\{C\left(\tau \right)-{\left[\phi \right]}^{2}\right\}$$ (52)

while the auto-correlation of the spikes term generates the instantaneous variability induced by the Poisson process:

$$\left[\left(\eta_{i}^{sp}\left(t\right)-\left[\eta_{i}^{sp}\right]\right)\left(\eta_{j}^{sp}\left(t+\tau \right)-\left[\eta_{j}^{sp}\right]\right)\right]=\delta_{ij}J^{2}\left(C_{E}+g^{2}C_{I}\right)\left[\phi \right]\delta \left(\tau \right)$$ (53)

By summing the two contributions together, and rescaling time appropriately, we obtain the evolution equation for Δ(*τ*) equivalent to Eq (48) with a self-consistent white noise term:

$$\ddot{\Delta }\left(\tau \right)=\Delta \left(\tau \right)-J^{2}\left(C_{E}+g^{2}C_{I}\right)\left\{C\left(\tau \right)-{\left[\phi \right]}^{2}+\frac{\left[\phi \right]}{\bar{\tau }}\delta \left(\tau \right)\right\}$$ (54)

#### Mean field theory in general EI networks

We discuss here the more general case of a block connectivity matrix, corresponding to one excitatory and one inhibitory population receiving statistically different inputs. The synaptic matrix is now given by:

$$\text{J}=J\left(\frac{{\text{J}}_{\text{EE}}|}{{\text{J}}_{\text{IE}}|}\frac{{\text{J}}_{\text{EI}}}{{\text{J}}_{\text{II}}}\right)$$ (55)

Each row of *J* contains exactly *C*_*E*_ non-zero excitatory entries in the blocks of the excitatory column, and exactly *C*_*I*_ inhibitory entries in the inhibitory blocks. Non-zero elements are equal to *j*_*E*_ in J_EE_, to −*g*_*E*_ *j*_*E*_ in J_EI_, to *j*_*I*_ in J_IE_, and to −*g*_*I*_ *j*_*I*_ in J_II_.

The network admits a fixed point $\left(x_{0}^{E},x_{0}^{I}\right)$ which is homogeneous within the two different populations:

$$\left(\begin{array}{c} x_{0}^{E}\\ x_{0}^{I} \end{array}\right)=J\left(\begin{array}{c} j_{E}\left(C_{E}\phi \left(x_{0}^{E}\right)-g_{E}C_{I}\phi \left(x_{0}^{I}\right)\right)\\ j_{I}\left(C_{E}\phi \left(x_{0}^{E}\right)-g_{I}C_{I}\phi \left(x_{0}^{I}\right)\right) \end{array}\right)$$ (56)

With linear stability analysis, we obtain that the fixed point stability is determined by the eigenvalues of matrix:

$$\text{S}=J\left(\frac{\phi_{E}^{\prime }{\text{J}}_{\text{EE}}|}{\phi_{E}^{\prime }{\text{J}}_{\text{IE}}|}\frac{\phi_{I}^{\prime }{\text{J}}_{\text{EI}}}{\phi_{I}^{\prime }{\text{J}}_{\text{II}}}\right)$$ (57)

where we used the short-handed notation $\phi_{k}^{\prime }=\phi^{\prime }\left(x_{0}^{k}\right)$.

The eigenspectrum of *S* consists of a densely distributed component, represented by a circle in the complex plane, and a discrete component, consisting of two outlier eigenvalues. The radius of the complex circle is determined by the 2x2 matrix containing the variance of the entries distributions in the four blocks, multiplied by *N* [12, 13, 34]:

$$\Sigma =J^{2}\left(\begin{array}{cc} {\phi_{E}^{\prime }}^{2}C_{E}j_{E}^{2} & {\phi_{I}^{\prime }}^{2}C_{I}g_{E}^{2}j_{E}^{2}\\ {\phi_{E}^{\prime }}^{2}C_{E}j_{I}^{2} & {\phi_{I}^{\prime }}^{2}C_{I}g_{I}^{2}j_{I}^{2} \end{array}\right)$$ (58)

More precisely, the radius of the circle is given by the square root of its larger eigenvalues:

$$\begin{array}{ll} r= & [\frac{1}{2}J^{2}\{C_{E}\phi_{E}^{\prime }{}^{2}j_{E}^{2}+C_{I}\phi_{I}^{\prime }{}^{2}g_{I}^{2}j_{I}^{2}\\ & {+\sqrt{{\left(C_{E}\phi_{E}^{\prime }{}^{2}j_{E}^{2}+C_{I}\phi_{I}^{\prime }{}^{2}g_{I}^{2}j_{I}^{2}\right)}^{2}-4C_{E}C_{I}\phi_{E}^{\prime }{}^{2}\phi_{I}^{\prime }{}^{2}j_{E}^{2}j_{I}^{2}\left(-g_{E}^{2}+g_{I}^{2}\right)}\}]}^{\frac{1}{2}} \end{array}$$ (59)

where the derivative terms *ϕ*′^*k*^ contain an additional dependency on *J*.

In order to determine the two outlier eigenvalues, we construct the 2x2 matrix containing the mean of *S* in each of the four blocks, multiplied by *N*:

$$M=J\left(\begin{array}{cc} \phi_{E}^{\prime }C_{E}j_{E} & -\phi_{I}^{\prime }C_{I}g_{E}j_{E}\\ \phi_{E}^{\prime }C_{E}j_{I} & -\phi_{I}^{\prime }C_{I}g_{I}j_{I} \end{array}\right)$$ (60)

The outliers correspond to the two eigenvalues of *M*, and are given by:

$$\xi_{\pm }=\frac{1}{2}J\left\{\phi_{E}^{\prime }C_{E}j_{E}-\phi_{I}^{\prime }C_{I}g_{I}j_{I}\pm \sqrt{{\left(\phi_{E}^{\prime }C_{E}j_{E}-\phi_{I}^{\prime }C_{I}g_{I}j_{I}\right)}^{2}+4\phi_{E}^{\prime }\phi_{I}^{\prime }C_{E}C_{I}j_{E}j_{I}\left(-g_{E}+g_{I}\right)}\right\}$$ (61)

Notice that, if *g*_*E*_ is sufficiently larger than *g*_*I*_, the outlier eigenvalues can be complex conjugates.

We focus on the case where, by increasing the global coupling *J*, the instability to chaos is the first bifurcation to take place. As in the simpler case when excitatory and inhibitory populations are identical, we need the real part of the outliers to be negative or positive but smaller than the radius *r* of the densely distributed component of the eigenspectrum. This requirement can be accomplished by imposing relative inhibitory strengths *g*_*E*_ and *g*_*I*_ strong enough to overcome excitation in the network. For a connectivity matrix which satisfies the conditions above, an instability to a fluctuating regime occurs when the radius *r* crosses unity.

We can use again DMF to analyze the network activity below the instability. To start with, dealing with continuous-time dynamics, one can easily generalize the mean field equations we recovered for the simpler two-column connectivity. In the new configuration, the aim of mean field theory is to determine two values of the mean activity and two values for the variance, one for each population.

By following the same steps as before, we define $\eta_{i}^{E}=\sum_{j=1}^{N}J_{ij}\phi \left(x_{j}\left(t\right)\right)$ for each *i* belonging to the *E* population, and $\eta_{i}^{E}=\sum_{j=1}^{N}J_{ij}\phi \left(x_{j}\left(t\right)\right)$ for each *i* belonging to *I*. Those two variables represent the effective stochastic inputs to excitatory or inhibitory units which replace the deterministic network interactions. Under the same hypothesis as before, we compute the statistics of the $\eta_{i}^{E}$ and $\eta_{i}^{I}$ distributions. For the mean, we find:

$$\left(\begin{array}{c} \left[\eta_{i}^{E}\right]\\ \left[\eta_{i}^{I}\right] \end{array}\right)=J\left(\begin{array}{cc} C_{E}j_{E} & -C_{I}g_{E}j_{E}\\ C_{E}j_{I} & -C_{I}g_{I}j_{I} \end{array}\right)\left(\begin{array}{c} \left[\phi^{E}\right]\\ \left[\phi^{I}\right] \end{array}\right)$$ (62)

For the second order statistics, we have:

$$\left(\begin{array}{c} \left[\left(\eta_{i}^{E}\left(t\right)-\left[\eta_{i}^{E}\right]\right)\left(\eta_{j}^{E}\left(t+\tau \right)-\left[\eta_{j}^{E}\right]\right)\right]\\ \left[\left(\eta_{i}^{I}\left(t\right)-\left[\eta_{i}^{I}\right]\right)\left(\eta_{j}^{I}\left(t+\tau \right)-\left[\eta_{j}^{I}\right]\right)\right] \end{array}\right)=J^{2}\left(\begin{array}{cc} C_{E}j_{E}^{2} & C_{I}g_{E}^{2}j_{E}^{2}\\ C_{E}j_{I}^{2} & C_{I}g_{I}^{2}j_{I}^{2} \end{array}\right)\left(\begin{array}{c} C^{E}\left(\tau \right)-{\left[\phi^{E}\right]}^{2}\\ C^{I}\left(\tau \right)-{\left[\phi^{I}\right]}^{2} \end{array}\right).$$ (63)

By using those results, we obtain two equations for the mean values of the input currents:

$$\left(\begin{array}{c} \mu^{E}\\ \mu^{I} \end{array}\right)=J\left(\begin{array}{cc} C_{E}j_{E} & -C_{I}g_{E}j_{E}\\ C_{E}j_{I} & -C_{I}g_{I}j_{I} \end{array}\right)\left(\begin{array}{c} \left[\phi^{E}\right]\\ \left[\phi^{I}\right] \end{array}\right).$$ (64)

and two differential equations for the auto-correlation functions, which can be summarized as:

$$\left(\begin{array}{c} {\ddot{\Delta }}^{E}\left(\tau \right)\\ {\ddot{\Delta }}^{I}\left(\tau \right) \end{array}\right)=\left(\begin{array}{c} \Delta^{E}\left(\tau \right)\\ \Delta^{I}\left(\tau \right) \end{array}\right)-J^{2}\left(\begin{array}{cc} C_{E}j_{E}^{2} & C_{I}g_{E}^{2}j_{E}^{2}\\ C_{E}j_{I}^{2} & C_{I}g_{I}^{2}j_{I}^{2} \end{array}\right)\left(\begin{array}{c} C^{E}\left(\tau \right)-{\left[\phi^{E}\right]}^{2}\\ C^{I}\left(\tau \right)-{\left[\phi^{I}\right]}^{2} \end{array}\right).$$ (65)

All the mean values are defined and computed as before, the population averages to be taken only over the *E* or the *I* population.

The main difficulty in solving Eqs (64) and (65) comes from the absence of an analogy with an equation of motion for a classical particle in a potential. Unfortunately, indeed, isolating the self-consistent solution in absence of an analogous suitable potential *V*(Δ^*E*^(*τ*), Δ^*I*^(*τ*)) appears to be computationally very costly.

However, if we restrict ourselves to discrete-time rate dynamics:

$$x_{i}\left(t+1\right)=\sum_{j=1}^{N}J_{ij}\phi \left(x_{j}\left(t\right)\right).$$ (66)

DMF equations can still easily be solved. With discrete-time evolution, the mean field dynamics reads:

$$x_{i}\left(t+1\right)=\eta_{i}\left(t\right)$$ (67)

which identifies directly the input current variable *x*_*i*_ with the stochastic process *η*_*i*_. In contrast to the continuous case, where self-consistent noise is filtered by a Langevin process, the resulting dynamics is extremely fast. As a consequence, the statistics of *η*_*i*_ directly translates into the statistics of *x*. We are left with four variables, to be determined according to four equations, which can be synthesized in the following way:

$$\left(\begin{array}{c} \mu_{E}\\ \mu_{I} \end{array}\right)=J\left(\begin{array}{cc} C_{E}j_{E} & -C_{I}g_{E}j_{E}\\ C_{E}j_{I} & -C_{I}g_{I}j_{I} \end{array}\right)\left(\begin{array}{c} \left[\phi^{E}\right]\\ \left[\phi^{I}\right] \end{array}\right).$$ (68)

$$\left(\begin{array}{c} \Delta_{0}^{E}\\ \Delta_{0}^{I} \end{array}\right)=J^{2}\left(\begin{array}{cc} C_{E}j_{E}^{2} & C_{I}g_{E}^{2}j_{E}^{2}\\ C_{E}j_{I}^{2} & C_{I}g_{I}^{2}j_{I}^{2} \end{array}\right)\left(\begin{array}{c} \left[\phi^{E2}\right]-{\left[\phi^{E}\right]}^{2}\\ \left[\phi^{I2}\right]-{\left[\phi^{I}\right]}^{2} \end{array}\right).$$ (69)

As usual, firing rate statistics are computed as averages with respect to a Gaussian distribution with mean *μ*_*E*_ (*μ*_*I*_) and variance $\Delta_{0}^{E}$ ($\Delta_{0}^{I}$).

When adopting discrete-time dynamics, a second condition has to be imposed on the connectivity matrix. To prevent phase-doubling bifurcations specific to discrete-time dynamics, we need the real part of the outliers to be strictly smaller than *r* in modulus. An isolated outlier on the negative real axis, indeed, would lose stability and induce fast oscillations in the activity before the transition to chaos takes place. The latter condition is satisfied in a regime where inhibition is only weakly dominating, coinciding with the phase region where the approximation provided by DMF is very good (see below in Methods).

#### Mean field theory with stochastic in-degree

We derive here the dynamical mean field equations for network in which the total number of inputs *C* varies randomly between different units in the network. We focus on a connectivity matrix with one excitatory and one inhibitory column. In the excitatory column, each element *J*_*ij*_ is drawn from the following discrete distribution:

$$J_{ij}=\left\{\begin{array}{cc} J & p=C_{E}/N_{E}=C/N\\ 0 & \text{otherwise} \end{array}\right.$$

Up to the order *O*(1/*N*), the statistics of the entries *J*_*ij*_ are are:

$$\left\langle J_{ij}\right\rangle =\frac{J}{N}C$$ (70)

$$\left\langle J_{ij}^{2}\right\rangle =\frac{J^{2}}{N}C.$$ (71)

The inhibitory column is defined in a similar way, if substituting *J* with −*gJ*.

We proceed in the same order as in the previous sections. We define the effective stochastic coupling, given by *η*_*i*_(*t*) = ∑_*j*_ *J*_*ij*_*ϕ*(*x*_*j*_(*t*)). We compute the equations for the mean and the correlation of the Gaussian noise *η*_*i*_ in the thermodynamic limit.

We will find that the variance associated to the single neuron activity will consist of a temporal component, coinciding with the amplitude squared of chaotic fluctuations, and of a quenched term, which appears when sampling different realizations of the random connectivity matrix.

For a given realization and a given unit *i*, the temporal auto-correlation coincides with: ${\left[\eta_{i}\left(t\right)\eta_{i}\left(t+\tau \right)\right]}_{t,ic}-{\left[\eta_{i}\right]}_{t,ic}^{2}$ by averaging over time and over different initial conditions. In a second step, averaging over all the units in the population, or equivalently, over the realizations of the matrix *J*_*ij*_, returns the average size of deviations from single unit mean within one single trial ${\left[{\left[\eta_{i}\left(t\right)\eta_{i}\left(t+\tau \right)\right]}_{t,ic}-{\left[\eta_{i}\right]}_{t,ic}^{2}\right]}_{J}=\left[\eta_{i}\left(t\right)\eta_{i}\left(t+\tau \right)\right]-{\left[{\left[\eta_{i}\right]}_{t,ic}^{2}\right]}_{J}$. Remember that, in our notation, [] indicates an average over time, initial conditions, and matrix realizations. One can compute self-consistently this quantity and check that it coincides with the expression for the total second order moment we found in the previous paragraph for the fixed in-degree case.

In order to close the expression for the DMF equations, we will need to express all the averages of *ϕ* in terms of the total variance Δ_0_, which includes quenched variability. For this reason, we compute the average deviations from [*η*_*i*_(*t*)*η*_*i*_(*t* + *τ*)] with respect to the population (realizations) mean [*η*_*i*_]. As a result, the second moment [*η*_*i*_(*t*)*η*_*j*_(*t* + *τ*)] − [*η*_*i*_(*t*)]^2^ will now include the static trial-to-trial variability.

For the mean, we get:

$$\begin{array}{cc} \left[\eta_{i}\left(t\right)\right] & =\left\langle \sum_{j_{E}=1}^{N_{E}}J_{ij_{E}}\phi \left(x_{j_{E}}\left(t\right)\right)\right\rangle +\left\langle \sum_{j_{I}=1}^{N_{I}}J_{ij_{I}}\phi \left(x_{j_{I}}\left(t\right)\right)\right\rangle =\left(N_{E}\left\langle J_{ij_{E}}\right\rangle +N_{I}\left\langle J_{ij_{I}}\right\rangle \right)\left\langle \phi \right\rangle \\ & =J\left(C_{E}-gC_{I}\right)\left\langle \phi \right\rangle . \end{array}$$ (72)

Applying the same steps as before, we compute the second order statistics:

$$\begin{array}{cc} & \left[\eta_{i}\left(t\right)\eta_{i}\left(t+\tau \right)\right]=\left\langle \sum_{k=1}^{N}J_{ik}\phi \left(x_{k}\left(t\right)\right)\sum_{l=1}^{N}J_{il}\phi \left(x_{l}\left(t+\tau \right)\right)\right\rangle \\ & =\left\langle \sum_{k_{E}=1}^{N_{E}}J_{ik_{E}}\phi \left(x_{k_{E}}\left(t\right)\right)\sum_{l_{E}=1}^{N_{E}}J_{il_{E}}\phi \left(x_{l_{E}}\left(t+\tau \right)\right)\right\rangle +\left\langle \sum_{k_{I}=1}^{N_{I}}J_{ik_{I}}\phi \left(x_{k_{I}}\left(t\right)\right)\sum_{l_{I}=1}^{N_{I}}J_{il_{I}}\phi \left(x_{l_{I}}\left(t+\tau \right)\right)\right\rangle \\ & +2\langle \sum_{k_{E}=1}^{N_{E}}J_{ik_{E}}\phi \left(x_{k_{E}}\left(t\right)\right)\sum_{l_{I}=1}^{N_{I}}J_{il_{I}}\phi \left(x_{l_{I}}\left(t+\tau \right)\right)\rangle . \end{array}$$ (73)

Again, we consider separate contributions from diagonal (*k* = *l*) and off-diagonal (*k* ≠ *l*) terms. This results in:

$$\begin{array}{cc} & \left[\eta_{i}\left(t\right)\eta_{i}\left(t+\tau \right)\right]=C_{E}J^{2}\left\langle \phi \left(x_{i}\left(t\right)\right)\phi \left(x_{i}\left(t+\tau \right)\right)\right\rangle +C_{E}^{2}\left(1-1/N_{E}\right)J^{2}{\left\langle \phi \right\rangle }^{2}\\ & -2C_{E}C_{I}gJ^{2}{\left\langle \phi \right\rangle }^{2}+C_{I}g^{2}J^{2}\left\langle \phi \left(x_{i}\left(t\right)\right)\phi \left(x_{i}\left(t+\tau \right)\right)\right\rangle +C_{I}^{2}\left(1-1/N_{I}\right)g^{2}J^{2}{\left\langle \phi \right\rangle }^{2}. \end{array}$$ (74)

As we can see, diagonal terms behave, on average, like in the fixed in-degree case. To estimate the off-diagonal contributions, we observe that for every *k*_*E*_ index, the expected number of other non-zero incoming connections is *C*_*E*_(1 − 1/*N*_*E*_). As a consequence, the *k*_*E*_ ≠ *l*_*E*_ sum contains on average $C_{E}^{2}$ terms of value *J*^2^〈*ϕ*〉^2^ in the limit *N* → ∞. Note that in the fixed in-degree case, the same sum contained exactly *C*_*E*_(*C*_*E*_ − 1) terms. That resulted in a smaller value for the second order statistics, which does not include the contribution from stochasticity in the number of incoming connections. Similar arguments hold for the inhibitory units.

To conclude, in the large network limit, we found:

$$\begin{array}{cc} & \left[\eta_{i}\left(t\right)\eta_{i}\left(t+\tau \right)\right]=J^{2}\left(C_{E}+g^{2}C_{I}\right)\left\langle \phi \left(x_{i}\left(t\right)\right)\phi \left(x_{i}\left(t+\tau \right)\right)\right\rangle +J^{2}{\left(C_{E}-gC_{I}\right)}^{2}{\left\langle \phi \right\rangle }^{2} \end{array}$$ (75)

such that the final result reads:

$$\left[\eta_{i}\left(t\right)\eta_{i}\left(t+\tau \right)\right]-{\left[\eta_{i}\left(t\right)\right]}^{2}=J^{2}\left(C_{E}+g^{2}C_{I}\right)C\left(\tau \right).$$ (76)

As before, one can then check that the cross-correlation between different units vanishes.

The noise distribution determines the following self-consistent potential:

$$V\left(\Delta ,\Delta_{0}\right)=-\frac{\Delta^{2}}{2}+J^{2}\left(C_{E}+g^{2}C_{I}\right)\int Dz{\left[\int Dx\Phi (\mu +\sqrt{\Delta_{0}-\Delta }x+\sqrt{\Delta }z)\right]}^{2}.$$ (77)

In contrast with the potential of Eq (35), which was found for networks with fixed in-degree, here we observe the lack of the term −Δ[*ϕ*]^2^. As a consequence, the new potential is flat around a non-zero Δ = Δ_∞_ value, which represents the asymptotic population disorder.

As usually, we derive the DMF solution in the weak and in the strong coupling regime thanks to the analogy with the one-dimensional equation of motion. When *J* < *J*_*C*_, the potential has the shape of a concave parabola, the vertex of which is shifted to Δ_∞_ ≠ 0. The only admittable physical solution is here Δ(*τ*) = Δ_0_ = Δ_∞_. In order to determine its value, we use the condition emerging from setting $\ddot{\Delta }=0$:

$$\Delta_{0}=J^{2}\left(C_{E}+g^{2}C_{I}\right)\int Dz\phi^{2}\left(\mu +\sqrt{\Delta_{0}}z\right)$$ (78)

to be solved together with the equation for the mean:

$$\mu =J\left(C_{E}-gC_{I}\right)\int Dz\phi \left(\mu +\sqrt{\Delta_{0}}z\right).$$ (79)

When *J* > *J*_*C*_, the auto-correlation acquires a temporal structure. The stable solution is monotonically decreasing from Δ_0_ to a value Δ_∞_, and we need to self-consistently determine *μ*, Δ_∞_ and Δ_0_ through three coupled equations. Apart from the usual one for *μ*, a second equation is given by the energy conservation law:

$$V\left(\Delta_{0},\Delta_{0}\right)=V\left(\Delta_{\infty },\Delta_{0}\right)$$ (80)

which reads:

$$\begin{array}{cc} \frac{\Delta_{0}^{2}-\Delta_{\infty }^{2}}{2}=J^{2}\left(C_{E}+g^{2}C_{I}\right) & \left\{\int Dz\Phi^{2}\left(\mu +\sqrt{\Delta_{0}}z\right)\right.\\ & \left.-\int Dz{\left[\int Dy\Phi (\mu +\sqrt{\Delta_{0}-\Delta_{\infty }}y+\sqrt{\Delta_{\infty }}z)\right]}^{2}\right\}. \end{array}$$ (81)

The third equation emerges from setting $\ddot{\Delta }=0$ at Δ_∞_, which gives:

$$\Delta_{\infty }=J^{2}\left(C_{E}+g^{2}C_{I}\right)\int Dz{\left[\int Dy\phi (\mu +\sqrt{\Delta_{0}-\Delta_{\infty }}y+\sqrt{\Delta_{\infty }}z)\right]}^{2}.$$ (82)

### Finite size effects and limits of the mean field assumptions

We test numerically the validity of the Gaussian assumptions and the predictions emerging from the DMF theory. We found two main sources of discrepancies between the theory and numerics, namely finite-size effects and the asymmetry between excitation and inhibition.

**Fig 11:**
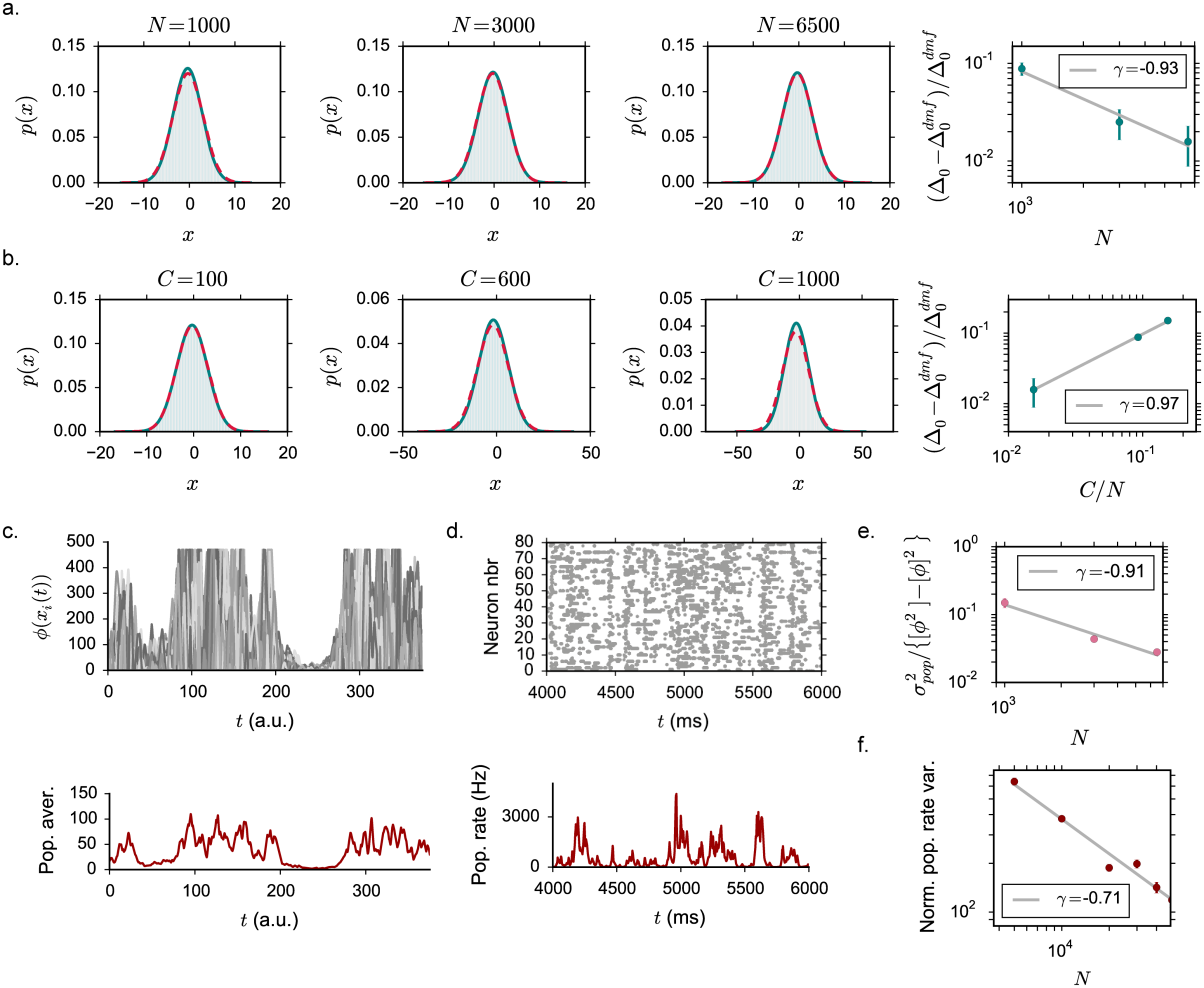
Comparison between dynamical mean field predictions and numerical simulations: Finite size effects. **a.** Dependence on the system size *N* (*C* = 100). In the first three panels: distribution of the input current *x* in the population and in different time steps. The numerical distribution is obtained through averaging over 3 realizations of the synaptic matrix. Light green: simulated data distribution, dark green: best Gaussian fit to data, red: DMF prediction. In the fourth panel: normalized deviations from the DMF theoretical value. The log-log dependence is fitted with a linear function, *γ* giving the coefficient of the linear term. Choice of the parameters: *g* = 4.1, *J* = 0.2, *ϕ*_*max*_ = 2. **b.** As in **a**, dependence on the in-degree *C* (*N* = 6500). **c.** Finite size effects in rate networks with large saturation upper-bound: sample of network activity (top: single units in grey scale, bottom: population averaged firing rate). Choice of the parameters: *g* = 5, *J* = 0.14, *ϕ*_*max*_ = 240. **d.** Finite size effects in networks of LIF neurons with small refractory period: sample of network activity (rastergram of 80 randomly selected neurons, population averaged firing rate). Choice of the parameters: *N* = 20000, *C* = 500, *g* = 5, *τ*_*rp*_ = 0.01 ms, *J* = 0.9 mV. **e.** Finite size effects in rate networks with large saturation upper-bound: normalized variance of the population-averaged firing rate as a function of the network size. **f.** Finite size effects in networks of LIF neurons with small refractory period: normalized variance of the population-averaged firing rate as a function of the network size (computed with 1 ms bins).

As a first step, we analyzed the magnitude of finite size effects deriving from taking finite network sizes. Fig 11a shows a good agreement between simulated data and theoretical expectations. The magnitude of finite size effects shrinks as the network size is increased and cross-correlations between different units decays.

In Fig 11b we tested instead the effect of increasing the in-degree *C* when *N* is kept fixed. When *C* is constant and homogeneous in the two populations, our mean field approach requires network sparseness (*C* ≪ *N*). Consistently, we find an increase in the deviations from the theoretical prediction when *C* is increased.

Both the *N* and *C* dependencies have the effect of weakly reducing fluctuations variance with respect to the one expected in the thermodynamic limit. The numerically obtained *x* distribution is in good agreement with the assumption of DMF, which states that current variables *x*_*i*_ are distributed, for large time *t* and size *N*, according to a Gaussian distribution of mean *μ* and variance Δ_0_.

We observe that stronger deviations from the theoretical predictions can arise when the upper-bound *ϕ*_*max*_ on the transfer function is large and the network is in the intermediate and strong coupling regime. By simulating the network activity in that case, we observe stronger cross-correlations among units, which can cause larger fluctuations in the population-averaged firing rate.

In Fig 11c we check that those deviations can still be understood as finite size effects: the distance between the DMF value and the observed ones, which now is larger, decreases with *N* as the correlation among units decay. Equivalently, the variance of the fluctuations in the population-averaged input current and firing rate decays consistently as ∼1/*N*.

The same effect, and even stronger deviations, are observed in rate models where the transfer function is chosen to mimic LIF neurons.

As a side note, we remark that strong correlations in numerical simulations are observed also in the case of spiking networks of LIF neurons with small refractory period and intermediate coupling values (Fig 11d). Also in this case, correlations are reflected in strong time fluctuations in the population averaged firing rate. Their amplitude should scale with the system size as 1/*N* in the case of independent Poisson processes. This relationship, which is well fitted in the weak and strong coupling regimes, appears to transform into a weaker power law decay for intermediate *J* values.

#### Limits of the Gaussian approximation

**Fig 12:**
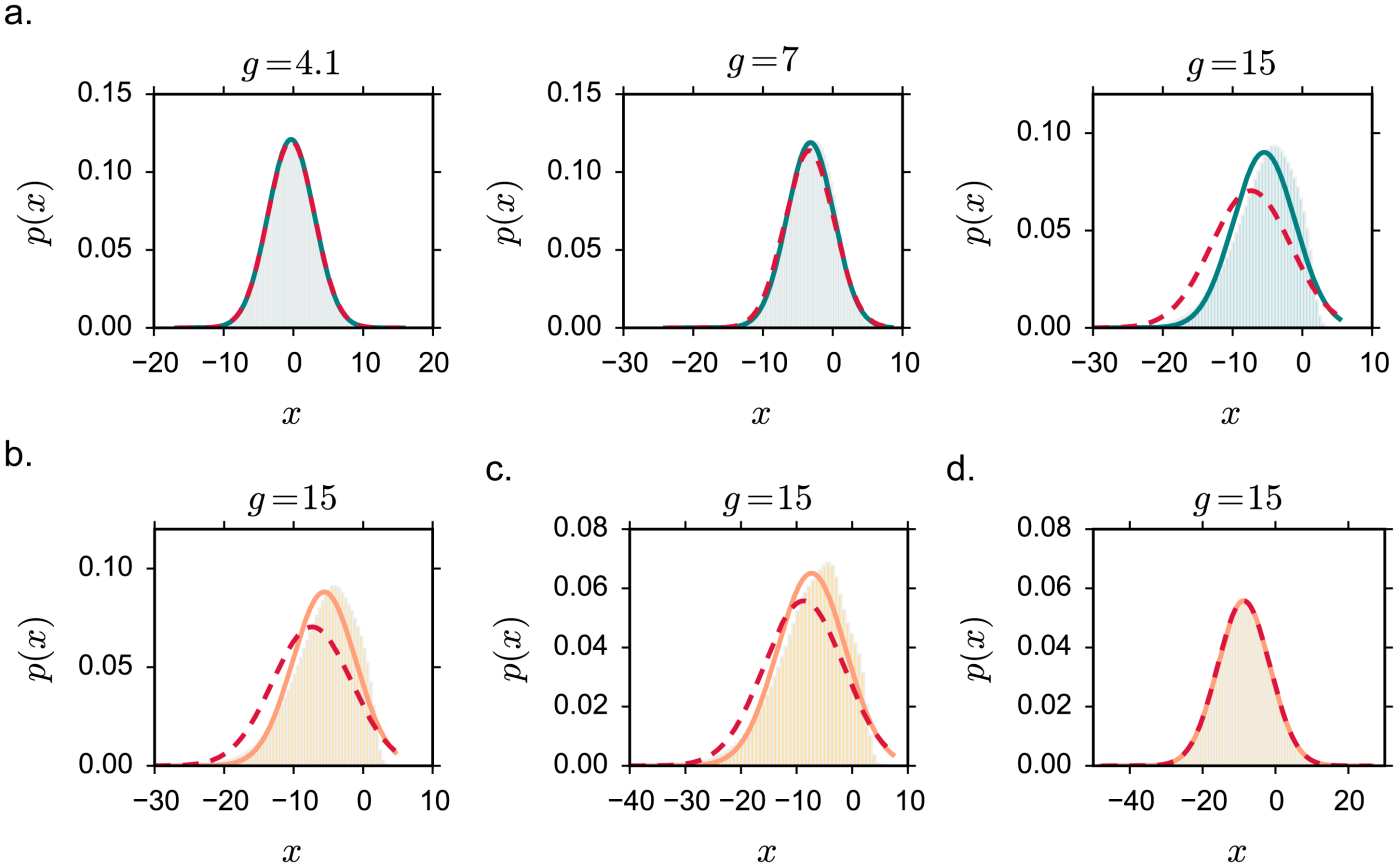
Comparison between dynamical mean field predictions and numerical simulations: The effects of strong inhibition. Distribution of the input current *x* in the population and in different time steps. The numerical distribution is obtained through averaging over 3 realizations of the synaptic matrix. Light green/orange: simulated data distribution, dark green/orange: best Gaussian fit to data, red: DMF prediction. Choice of the parameters: *C* = 100, *N* = 6500, *J* = 0.2. **a.** Dependence on the inhibition dominance *g*. **b.** Numerical distribution for a network with a synaptic matrix where *C* is fixed, as above, but excitatory and inhibitory units are shuffled. **c.** As above, with a synaptic matrix where *C* is random. **d.** As above, with the equivalent Gaussian matrix, whose statistics match the ones of the sparse one.

A different effect is found by increasing the dominance of inhibition over excitation in the network, i.e. by increasing *g*, or equivalently, by decreasing *f*. As shown in Fig 12a, inhibition dominance can significantly deform the shape of the distribution, which displays suppressed tails for positive currents. As the inhibition dominance is increased, since *ϕ*(*x*_*i*_) is positive and *J*_*ij*_ strongly negative on average, the fluctuations become increasingly skewed in the negative direction. As expected, the Gaussian approximation does not fit well the simulated data. Fig 11b, 11c and 11d shows that the same effect is quite general and extends to networks where excitation and inhibition are not segregated or the connectivity *C* is random.

An extreme consequence of this effect is the failure of DMF in describing purely inhibitory networks in absence of external excitatory currents, where the effective coupling *η*_*i*_(*t*) = ∑_*j*_ *J*_*ij*_*ϕ*(*x*_*j*_(*t*)) is strictly non-positive at all times. In this case, DMF erroneously predicts a critical coupling *J*_*D*_ between a bounded and an unbounded regime, the divergence being led by the positive tails of the Gaussian bell. In contrast, in absence of any positive feedback, purely inhibitory networks cannot display a transition to run-away activity.

As a final remark, we observe that the agreement between simulated activity and mean field predictions in the case of purely inhibitory networks is in general less good than the one we found for EI architectures.

We conclude that the Gaussian hypothesis adopted in the DMF framework is a reasonable approximation only when inhibition does not overly dominate excitation. Finally, we remark that this limitations critically depends on adopting sparse matrices where non-zero entries have fixed values. If adopting a Gaussian, fully-connected connectivity, whose mean and variance are matching the ones of the original matrix:

$$\begin{array}{cc} & \left[J_{ij}\right]=\frac{J}{N}\left(C_{E}-gC_{I}\right)\\ & \left[J_{ij}^{2}\right]=\frac{J^{2}}{N}\left(C_{E}+g^{2}C_{I}\right) \end{array}$$ (83)

numerical simulations reveal that, whatever the degree of inhibition, positive entries are strong enough to balance the distribution, which strongly resembles again a Gaussian bell.

### Network of integrate-and-fire neurons

The simulations presented in Fig 9 were performed on a network of leaky integrate-and-fire (LIF) neurons identical to [19]. The membrane potential dynamics of the *i*-th LIF neuron are given by:

$$\tau_{m}\frac{dV_{i}}{dt}=-V_{i}+\mu_{0}+RI_{i}\left(t\right)+\mu_{\text{ext}}\left(t\right)$$ (84)

where *τ*_*m*_ = 20 ms is the membrane time constant, *μ*_0_ is a constant offset current, and *RI*_*i*_ is the total synaptic input from within the network. When the membrane potential crosses the threshold *V*_*th*_ = 20 mV, an action potential is emitted and the membrane potential is reset to the value *V*_*r*_ = 10 mV. The dynamics resume after a refractory period *τ*_*r*_, the value of which was systematically varied. The total synaptic input to the i-th neuron is:

$$\begin{array}{c} RI_{i}\left(t\right)=\tau_{m}\sum_{j}J_{ij}\sum_{k}\delta \left(t-t_{j}^{\left(k\right)}-\Delta \right) \end{array}$$ (85)

where *J*_*ij*_ is the amplitude of the post-synaptic potential evoked in neuron *i* by an action potential occurring in neuron *j*, and Δ is the synaptic delay (here taken to be 1.1 ms). Note that if the synaptic delay is shorter than the refractory period, the network develops spurious synchronization [21].

The connectivity matrix *J*_*ij*_ was identical to the rate network with fixed in-degree described above.
